# Eggshell Porosity Provides Insight on Evolution of Nesting in Dinosaurs

**DOI:** 10.1371/journal.pone.0142829

**Published:** 2015-11-25

**Authors:** Kohei Tanaka, Darla K. Zelenitsky, François Therrien

**Affiliations:** 1 Department of Geoscience, University of Calgary, Calgary, Alberta, Canada; 2 Royal Tyrrell Museum of Palaeontology, Drumheller, Alberta, Canada; University of Akron, UNITED STATES

## Abstract

Knowledge about the types of nests built by dinosaurs can provide insight into the evolution of nesting and reproductive behaviors among archosaurs. However, the low preservation potential of their nesting materials and nesting structures means that most information can only be gleaned indirectly through comparison with extant archosaurs. Two general nest types are recognized among living archosaurs: 1) covered nests, in which eggs are incubated while fully covered by nesting material (as in crocodylians and megapodes), and 2) open nests, in which eggs are exposed in the nest and brooded (as in most birds). Previously, dinosaur nest types had been inferred by estimating the water vapor conductance (i.e., diffusive capacity) of their eggs, based on the premise that high conductance corresponds to covered nests and low conductance to open nests. However, a lack of statistical rigor and inconsistencies in this method render its application problematic and its validity questionable. As an alternative we propose a statistically rigorous approach to infer nest type based on large datasets of eggshell porosity and egg mass compiled for over 120 extant archosaur species and 29 archosaur extinct taxa/ootaxa. The presence of a strong correlation between eggshell porosity and nest type among extant archosaurs indicates that eggshell porosity can be used as a proxy for nest type, and thus discriminant analyses can help predict nest type in extinct taxa. Our results suggest that: 1) covered nests are likely the primitive condition for dinosaurs (and probably archosaurs), and 2) open nests first evolved among non-avian theropods more derived than *Lourinhanosaurus* and were likely widespread in non-avian maniraptorans, well before the appearance of birds. Although taphonomic evidence suggests that basal open nesters (i.e., oviraptorosaurs and troodontids) were potentially the first dinosaurs to brood their clutches, they still partially buried their eggs in sediment. Open nests with fully exposed eggs only became widespread among Euornithes. A potential co-evolution of open nests and brooding behavior among maniraptorans may have freed theropods from the ground-based restrictions inherent to covered nests and allowed the exploitation of alternate nesting locations. These changes in nesting styles and behaviors thus may have played a role in the evolutionary success of maniraptorans (including birds).

## Introduction

Nests are varied structures that play an important role in archosaur biology because they are used for incubating eggs and, in many species, for raising young. The nests can consist of simple scrapes or holes in the ground, bowl-shaped structures, or large vegetation mounds [1,2], and their architecture is suited not only for the incubation of eggs in a given environment but also for the incubation behavior/method of a species. Among extant archosaurs, two general types of nest are observed: 1) covered nests, in which the eggs are covered by organic/inorganic matter, are built by species that incubate their eggs using external heat sources (e.g., solar heat, plant decomposition, or geothermal heat [3]), and 2) open nests, in which the eggs are not covered by substrate and left exposed, are built by species that brood their eggs. Because all crocodylian species build covered nests and all bird species, except those of megapodes, incubate eggs in open nests [4], the transition from covered to open nest type likely occurred among non-avian dinosaurs (e.g., [5]).

Nest types and associated nesting behaviors are poorly understood in extinct archosaurs, including non-avian dinosaurs, in part because nest structures and nesting materials are rarely preserved [6,7]. Even on the rare occasions where nest structures are found (e.g., excavations, mounds; [7–10]), there is no indication of whether the eggs were covered by organic/inorganic material or surrounded by nesting materials as typically found in living archosaurs. Consequently, other evidence related to egg clutches, such as their taphonomic and sedimentologic setting or eggshell structures (i.e., pore canals), have been used to infer the nest type of dinosaurs (e.g., [7,11]).

Most prior studies have used a method that estimates the diffusive capacity of the eggshell, referred to as water vapor conductance (i.e.,G_H2O_), to infer the types of nest built by dinosaurs. Water vapor conductance in living archosaurs has usually been measured experimentally via daily water loss of a fresh egg (e.g., [12,13]). A theoretical formula to calculate water vapor conductance from eggshell porosity was also developed based on Fick's law of diffusion (herein referred to as morphometric G_H2O_) [12]. This formula was used initially by Seymour [11] to calculate G_H2O_ for dinosaur eggs, and nest type was inferred on the premise that covered nests are found in living species with high G_H2O_ and open nests in species with low G_H2O_ values. Thus, morphometric G_H2O_ values of dinosaurs were compared directly with experimental G_H2O_ values of living archosaurs (e.g., [11,14–17]), although the latter were calculated from measurement of daily water loss of an egg (i.e., experimental G_H2O_) and not from the theoretical formula (i.e., morphometric G_H2O_). However, a recent study compared morphometric and experimental G_H2O_ values in living archosaur species, and demonstrated that these two datasets/methods are mutually incongruent, likely due to systematic errors [18]. Thus direct comparison between morphometric and experimental G_H2O_ values, as widely applied to infer nest type of dinosaurs, may not be valid.

As a viable alternative to the water vapor conductance method, we present a statistically rigorous approach using eggshell porosity in order to predict nest type in extinct archosaurs. We apply this approach to the eggs of a variety of dinosaurs, including titanosaurs, the theropod *Lourinhanosaurus*, oviraptorosaurs, and troodontids, in order to assess their nesting habits and discuss the evolution of nest type and incubation behaviors among archosaurs.

## Material and Methods

A series of methodological steps were taken to document the relationship between eggshell porosity and nest types in crocodylians and birds in order to infer nest types in extinct archosaurs. Data on eggshell porosity (A_p_∙L_s_^-1^, in mm), egg mass (M, in g), and nest types (see "Nest classification for extant taxa") were compiled for living crocodylians and birds and subsequently compared statistically to test whether eggshell porosity relative to egg mass differs between open and covered nest types. Eggshell porosity and egg mass were then estimated for a variety of extinct archosaurs, including crocodylomorphs, non-avian dinosaurs, and birds. Through comparison with the extant dataset, discriminant analyses were used to infer nest types in extinct taxa. Both phylogenetic and non-phylogenetic (i.e., conventional) approaches were applied for the statistical analyses.

### Relationship between Water Vapor Conductance and Eggshell Porosity

Water vapor conductance of living species has usually been measured experimentally (e.g., [12,13]), but it has also been shown to be related to the geometry of eggshell pore canals. Ar et al. [12] were the first to derive a mathematical equation to calculate morphometric water vapor conductance (G_H2O_) using pore geometry in archosaur eggs. This equation is expressed as:

$$G_{H2O}=\frac{c\times D_{H2O}}{R\times T}\times \frac{A_{p}}{L_{s}}$$ (1)

where c is a unit conversion constant (1.56 x 10^9^ mgH_2_O∙s∙day^-1^∙mol^-1^), D_H2O_ is the diffusion coefficient of water vapor (mm^2^∙s^-1^) in air, R is the universal gas constant (6.24 x 10^7^ mm^3^∙torr∙mol^-1^°K^-1^), T is the absolute temperature of incubation (°K), A_p_∙L_s_^-1^ is eggshell porosity, A_p_ is the total pore area of an egg (mm^2^), and L_s_ is pore length (mm) [12]. Since many variables (i.e., c, D_H2O_, R, and T) can be safely assumed to be consistent among species (e.g., [12,13,18]), the equation can be simplified and expressed as G_H2O_ = 2.1∙A_p_∙L_s_^-1^ (see [18]). Morphometric water vapor conductance is thus directly proportional to eggshell porosity. Given that morphometric water vapor conductance (and hence eggshell porosity) is influenced by absolute nest humidity (S1 Text), which in turn is correlated with nest architecture or type (covered vs. open, see [19]), a correlation between eggshell porosity and nest types can be sought (see S1 Text for further explanation).

### Selection of extant taxa

Eggshell porosity, egg mass and nest type for 127 species of extant birds and crocodylians were gathered from either the literature (see [18]) or via new measurements of egg specimens (S2, S3 and S4 Tables). Egg specimens were permitted to be accessed from the institutions listed in S2 Table. The dataset includes only species with pore canals that approximate simple (unbranched) or tubular structures because porosity of eggshells with more complex pores (e.g., branched pores) could not be accurately estimated (e.g., *Casuarius*, *Dromaius*, *Pterocnemia*, *Rhea*, and *Struthio*; [20–23]). Although some pores of crocodylian eggshells may be irregularly shaped, they are usually simple and straight [24] and are here assumed to be tubular.

### Nest classification for extant taxa

Nest structures of extant archosaurs were classified into two general types, covered nests and open nests, based on information available in the literature (S4 Table). Covered nests are defined as those in which the eggs are completely covered with vegetation and/or sediment (e.g., mound or infilled hole nests on/in the ground), whereas open nests are those in which the eggs are partly or fully exposed, and may have nest materials surrounding a portion of the eggs (e.g., scrape, cup, plate, and dome nests) (see [19]).

Certain aquatic birds (e.g., Podicipediformes, *Cygnus*, *Oxyura*, *Chlidonias niger*, and *Gavia immer*) were excluded from this study due to their unusual nesting style. Because these birds build open nests floating on water with nest materials that can be wet [25–29], presumably resulting in high nest humidity [19,25–27], their eggshell porosity and water vapor conductance are anomalously high for birds with open nests [13,26,30].

### Selection of fossil eggs/ootaxa

Eggshell porosity and egg mass for 29 extinct archosaur taxa and ootaxa (i.e., egg taxa) were compiled from the literature or from new/additional measurements of egg specimens listed in S2 Table. Only species and oospecies with simple pore canals and for which data for individual pore area, pore density, pore length, and egg length and breadth were available were included in this study (Table 1). Eggs and ootaxa with complex or irregular pores {e.g., Dendroolithidae (*Torvosaurus* and possibly therizinosaur), Faveoloolithidae (?Sauropodomorpha), Ovaloolithidae (?Ornithopoda), and Spheroolithidae (*Maiasaura*-like eggs, presumably hadrosaur)} and taxa/ootaxa for which the data were potentially derived from the combination of multiple oospecies (e.g. *'Hypselosaurus'* and *'Protoceratops'* in [11] and Elongatoolithidae in [31,32]) were not included in this study. Also, eggshell porosity for enantiornithine eggs (e.g. '*Gobipteryx minuta*' in [31]) was not estimated in our study because the original article [31] indicated questionable values for both total number of pores and individual pore area.

**Table 1:** List of extinct archosaur taxa/ootaxa with estimated egg mass (M) and eggshell porosity (A_p_∙Ls-1) used in this study.

Family or oofamily(Possible taxonomic affinity)	Taxon or ootaxon	log M	log A_p_∙L_s_^-1^	Sources
Krokolithidae (Crocodylomorpha)	*Bauruoolithus fragilis*	1.599	1.687	Oliveira et al. [33]
Cairanoolithidae (Ornithischia?)/ Fusioolithidae	*Cairanoolithus dughii*	3.468	2.732	Williams et al. [14]; Garcia and Vianey-Liaud [34]
	*Cairanoolithus roussetensis*	3.430	2.761	Garcia and Vianey-Liaud [34]
Megaloolithidae (Sauropoda) / Fusioolithidae	*Megaloolithus aureliensis*	3.705	3.327	Garcia and Vianey-Liaud [34]
	*Megaloolithus mammilare*	3.716	3.055	Williams et al. [14]; Garcia and Vianey-Liaud [34]
	*Megaloolithus microtuberculata*	3.351	2.602	Garcia and Vianey-Liaud [34]
	*Megaloolithus patagonicus*/ titanosaur sauropod	3.107	2.703	Jackson et al. [16]; Grellet-tinner et al. [35]
	*Megaloolithus petralta*	3.420	2.788	Garcia and Vianey-Liaud [34]
	*Megaloolithus pseudomamillare*	3.550	2.842	Garcia and Vianey-Liaud [34]
	*Megaloolithus siruguei*	3.622	3.327	Williams et al. [14]; Lopez-Martinez et al. [36]; Garcia and Vianey-Liaud [34]; Deeming [15]; Jackson et al. [16]
	*Megaloolithus* cf. *siruguei*	3.325	3.138	Grigorescu et al. [37,38]
	*Megaloolithus* sp. (recrystallized)	3.267	2.548	Zelenitsky pers obs. (cited in [15])
	*Megaloolithus* sp. (non-recrystallized)	3.430	3.090	Zelenitsky pers obs. (cited in [15])
	Undetermined megaloolithid oospecies 1	3.235	3.085	Williams et al. [14]
	Undetermined megaloolithid oospecies 2	3.081	2.458	Grellet-tinner et al. [35]
Oofamily Indet. (Non-avian theropod)	*Continuoolithus canadensis*	2.320	1.785	Jackson et al. [39]
Allosauroidea?/ Coelurosauria?	*Lourinhanosaurus antunesi*	2.799	2.377	Antunes et al. [40]; Deeming [15]
Elongatoolithidae (Oviraptorosauria)	Elongatoolithus andrewsi*	2.584	1.621	Zhao [1975]; Mou [41]
	*Elongatoolithus elongatus*	2.411	1.659	Zhao et al. [42]
	*Macroelongatoolithus xixiaensis*	3.488	2.415	Zelenitsky pers obs. (cited in [15])
	Macroolithus rugustus*	2.772	1.642	Zhao [43]; Mou [41]
	Macroolithus yaotunensis*/ oviraptorosaurs	2.911	1.835	Zhao [43]; Mou [41]; Wiemann et al. [44]
Prismatoolithidae (Non-oviraptorosaur maniraptoran)	*Prismatoolithus levis*/ *Troodon formosus*	2.463	1.213	Zelenitsky and Hills [45]; Varricchio et al. [17]; Zelenitsky pers obs. (cited in [15]; this study
	*Protoceratopsidovum fluxuosum*	2.411	1.602	"Ornamented protoceratopsid egg" in Sabath [31]
	*Protoceratopsidovum minimum*	2.106	1.523	"Thin-shelled protoceratopsid egg" in Sabath [31]
	*Protoceratopsidovum sincerum*	2.380	1.465	"Smooth-shelled protoceratopsid egg" in Sabath [31]
	*Sankofa pyrenaica*	1.788	0.478	Lopez-Martinez and Vicens [46]
Dinornithiformes (moas)	*Euryapteryx* sp.	2.771	1.765	Gill [47]; Huynen et al. [48]; this study
	*Pachyornis geranoides*	2.771	1.41	Gill [47]; Huynen et al. [48]; this study

Asterisk (*) indicates that eggshell thickness was taken from Zhao [43] because Mou [41] provided only the thickness of the continuous layer as pore length.

The taxonomic affinity of most ootaxa considered in this study is well-established, particularly at higher taxonomic levels. For example, the ootaxon *Bauruoolithus* is attributed to a crocodylomorph based on eggshell microstructure [33]. The ootaxon *Megaloolithus patagonicus* is referred to a titanosaur based on its association with embryonic remains [49,50], thus eggs of the Megaloolithidae oofamily are widely regarded as belonging to sauropods [49,51,52]. Formerly classified in Megaloolithidae, the ootaxon *Cairanoolithus* has recently been re-assigned to a new oofamily, Cairanoolithidae, by Sellés and Galobart [53] who suggested it may belong to an ornithischian dinosaur.

The taxonomic identity of some theropod eggs is also known based on association with either embryonic or parental skeletal remains. These include the eggs of *Lourinhanosaurus antunesi*, a large theropod of either allosauroid [54,55] or coelurosaurian [56] affinity, the ootaxon *Macroolithus yaotunensis*, assigned to an oviraptorosaur [57], and the ootaxon *Prismatoolithus levis*, assigned to *Troodon formosus* [58]. Elongatoolithid and prismatoolithid ootaxa are attributed to Oviraptorosauria [57,59–64] and non-oviraptorosaur maniraptorans [46,65], respectively, based on eggshell microstructure similarities with eggs of known taxonomic identity. The ootaxon *Continuoolithus* is assigned to an indeterminate theropod based on egg and eggshell morphology [9,39,66]. Moa eggshells have been assigned to two small-bodied (female body masses 20–30 kg) species, *Pachyornis geranoides* and *Euryapteryx* sp., based on DNA analyses [48].

### Eggshell porosity

**Fig 1:**
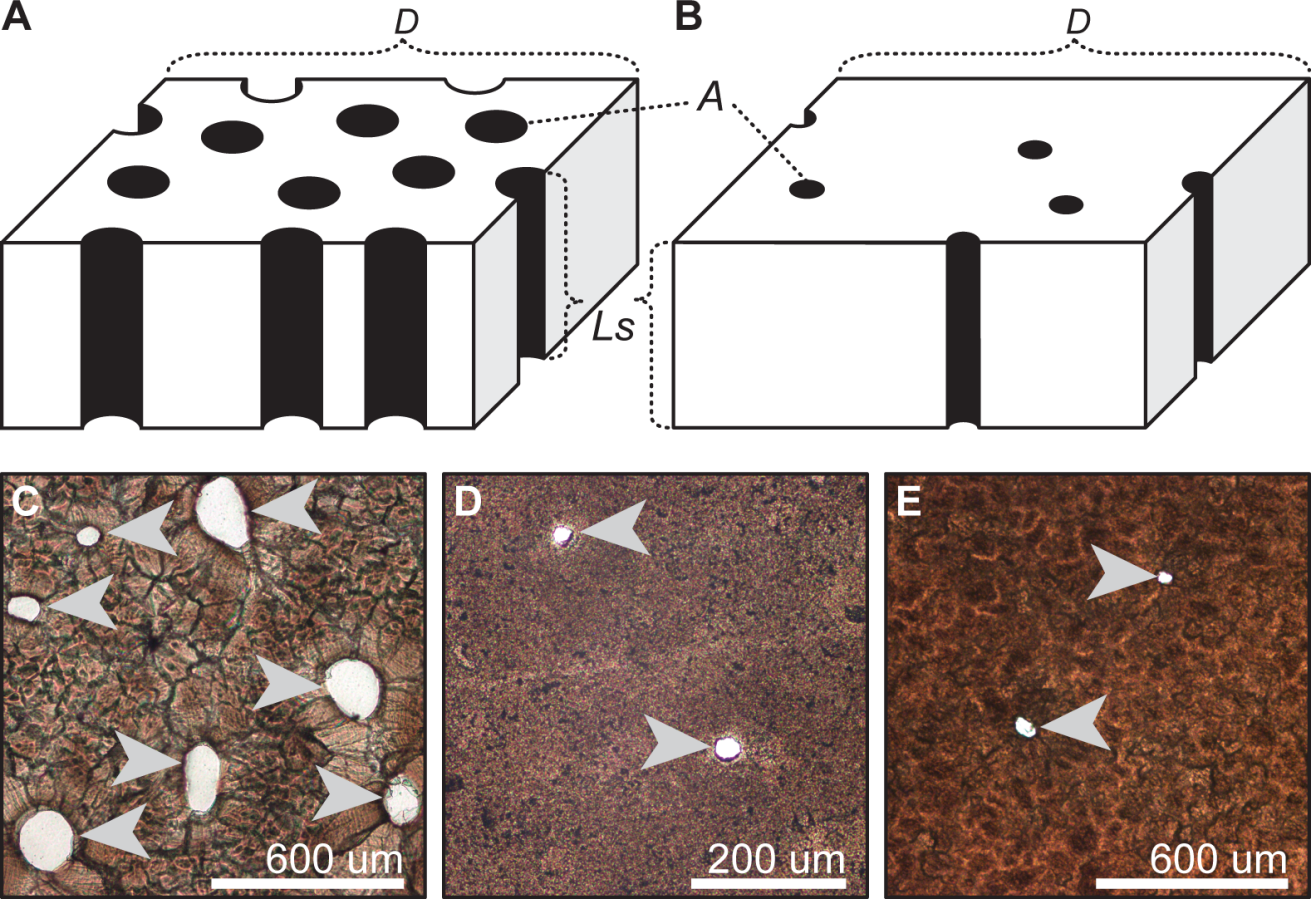
Porosity of archosaur eggshell. Schematic diagram of archosaur eggshell with high porosity (A) and low porosity (B), modified from [18]; tangential thin sections of living covered nester *Caiman latirostris* (C), living open nester *Pavo cristatus* (D), and non-avian maniraptoran *Troodon formosus* (E). Abbreviations; A, individual pore area; D, pore density; L_s_, pore length. Arrows indicate pore canals.

Eggshell porosity (A_p_∙L_s_^-1^, in mm) of both living and extinct archosaurs was determined by dividing the total pore area of an egg (A_p_, in mm^2^) by pore length (L_s_, in mm) (Tables 2 and 3; Fig 1A and 1B), except for *Macroolithus yaotunensis* by [44] where eggshell porosity was calculated based on its morphometric G_H2O_ value. Total pore area of an egg was calculated by multiplying individual pore area (A, in mm^2^) by pore density (D, in mm^-2^) and eggshell surface area (A_s_, in mm^2^). When pore density was not available in the literature, total pore area was calculated by multiplying individual pore area by the total number of pores in an egg (N). Because more than one value was usually available for each variable (i.e., A, D, L_s_, N, egg length, egg breadth), a mean value was calculated from the various sources/samples for each taxon/ootaxon.

**Table 2:** List of variables used for this study, modified from Tanaka and Zelenitsky [18].

Variable	Definition	Unit
A	Individual pore area	μm^2^
A_p_	Total pore area	mm^2^
A_p_∙L_s_^-1^	Eggshell porosity	mm
A_s_	Surface area of eggshell	mm^2^
B	Maximum egg breadth	mm
D	Pore density	mm^-2^
L	Maximum egg length	mm
L_s_	Shell thickness (= pore length)	mm
M	Egg mass	g
N	Total number of pores	per egg
V	Egg volume	mm^3^

**Table 3:** List of equations used for this study, modified from Tanaka and Zelenitsky [18].

Equation	Sources
A_p_ = A∙A_s_∙D = A∙N	Seymour [11]
A_s_ = 4.951∙V^0.666^	Paganelli et al. [67]
M = 5.48∙10^−4^∙LB^2^	Hoyt [68]
V = 0.51∙LB^2^	Hoyt [68]

For taxa/ootaxa where eggshell porosity values could not be obtained from the literature, they were calculated from the measurement of relevant variables in thin sections of eggshell specimens. Whenever possible, eggshell samples were taken from different regions of an egg (i.e., around equator and two poles) in order to capture regional variation. Shell thickness, which can be used as a proxy for pore length [12,13], was measured with a digital micrometer Mitutoyo CPM30-25MJ (precision = 2 μm). Individual pore area was measured from tangential thin sections of eggshells using a Leica DM2500P petrographic microscope (Fig 1C–1E) following the procedures of [18]. For pore counting in living birds, the inner surface of the eggshells was stained with methylene blue solution to accentuate the pores (see [13]). Pore density was estimated by counting the number of pore openings on the outer surface of eggshells using a Leica M80 binocular microscope, following the procedure of Tanaka and Zelenitsky [18]. Pore density in *Troodon* was estimated from tangential thin sections because the outer surface was poorly preserved. Eggshell surface area was calculated from maximum egg length (L, in mm) and breadth (B, in mm), both obtained from the literature, using the equations of Paganelli et al. [67] and Hoyt [68] (Tables 2 and 3), except for the ootaxa *Elongatoolithus andrewsi*, *Macroolithus rugustus*, and *M*. *yaotunensis* for which eggshell surface area were obtained from Mou [41]. Because egg length and breadth of the *Pachyornis geranoides* and *Euryapteryx* sp. specimens studied are unknown, these values were taken from intact eggs (AIM LB4003, LB4005, and an unregistered AIM egg of [47]) found at the same locality, which show comparable eggshell thickness and pore morphology to the specimens used in our study [69,70].

A possible caveat for the calculation of porosity in fossil eggshells is that diagenesis can alter pore dimensions [16,71]. Diagenetic dissolution for example can decrease pore length and enlarge pore canals, resulting in overestimation of eggshell porosity [16,71]. Because most values for fossil specimens were obtained from the literature it is impossible to assess the impact of diagenetic alteration on the ootaxa considered in this study. We proceed with the assumption that, overall, diagenesis did not significantly affect calculation of eggshell porosity.

### Egg mass

Mean egg mass (M, in g) for living and extinct archosaurs was compiled for this study. Egg mass for living species was obtained from the literature (S3 Table) and that for fossil taxa/ootaxa was estimated from egg length and breadth using the equation of Hoyt [68] (Table 3). Although other methods exist to estimate fossil egg mass (see [17]), they produce results that are consistent (within 10%) with Hoyt’s [68] method [17,39]. Therefore, for consistency, we applied Hoyt's method to all extinct ootaxa/taxa. Egg mass for *Elongatoolithus andrewsi*, *Macroolithus rugustus*, and *M*. *yaotunensis* was taken from Mou [41], who had used Hoyt's [68] method to derive his estimates.

### Phylogenetic distribution of nest type

The nature of the phylogenetic distribution (i.e., random vs. clumped) of nest types among living archosaurs was investigated based on our compiled extant dataset. Because nest type can be coded as a binary trait (covered vs. open), Fritz and Purvis' [72] D statistic was calculated by running 1000 permutations of the 'phylo.d' function of the package 'caper' using the software platform R3.1.3 (http://www.r-project.org/). For the D statistic, a value equal to or higher than 1.0 indicates a random phylogenetic distribution, whereas a value equal to or lower than 0 indicates a non-random phylogenetic distribution (i.e., phylogenetically clumped). The 'phylo.d' function provides *p* values to indicate whether the estimated D statistic is significantly different from 0 and 1, respectively.

The D statistic was run using a phylogenetic tree of 127 species of living birds and crocodylians compiled from the large-scale phylogeny of Jarvis et al. [73] and other publications for small-scale interrelationships (S3 Fig). Branch length was estimated from the divergence times of each node following the procedures of Motani and Schmitz [74] and Schmitz and Motani [75]. Divergence times of major clades were obtained from Time Tree (http://timetree.org) for birds and from Oaks [76] for crocodylians. Terminal taxon ages were set to zero. The phylogenetic tree and character matrix were constructed with the PDAP module v.1.16 [77] of the software Mesquite 3.02 [78].

### Analysis of covariance

Eggshell porosity relative to egg mass was compared between extant open-nesting and covered-nesting archosaurs using both conventional and phylogenetically-corrected analysis of covariance (ANCOVA and pcANCOVA, respectively). Non-phylogenetic, ordinary least-squares regression (OLS) was implemented for conventional ANCOVA with IBM SPSS Statistics v. 22.0.0 (IBM SPSS Inc.), whereas phylogenetically-corrected ANCOVA was implemented with the MATLAB (MathWorks Inc.) program Regressionv2.m (available upon request from T. Garland Jr.) following the method of Lavin et al. [79]. A phylogenetic variance-covariance matrix for Regressionv2.m was generated with the DOS PDDIST program [80]. Regressions for pcANCOVA were generated with two evolutionary models: regressions with Brownian motion (PGLS) and Ornstein-Uhlenbeck models (RegOU). PGLS assumes an evolutionary process with "random walk in continuous time" (e.g., [79]), whereas RegOU assumes an evolutionary process of "wandering back and forth on a selective peak" [79,81,82]. These three regression models (OLS, PGLS, and RegOU) were compared using the Akaike Information Criterion (AIC) to determine the best fit model of regression, where a lower AIC value indicates a better fit (e.g., [79,83,84]).

Nest type (open and covered nests) was considered a categorical variable, a covariate of egg mass, and a dependent variable of eggshell porosity in these analyses. Values of eggshell porosity (A_p_∙L_s_^-1^) and egg mass (M) were log-10 transformed prior to analysis. The normality and homogeneity of variances of the dataset were tested by non-phylogenetic Shapiro-Wilk tests and Levene tests using IBM SPSS Statistics v. 22.0.0. Residuals of log A_p_∙L_s_^-1^, calculated from OLS regressions for each nest type, were used for the Shapiro-Wilk tests.

The phylogenetic tree compiled for the D statistic (see above) was used for the pcANCOVA. In addition to the branch length determination method based on divergence time used for the D statistic, an arbitrary standardized method was also applied to assign branch length for the pcANCOVA because branches were not adequately standardized by divergence time. An arbitrary branch length model was used by following the procedure of Garland et al. [85], resulting in all branch lengths equal to one.

### Discriminant analysis

Nest type of fossil taxa/ootaxa was inferred by analyzing their eggshell porosity using conventional, non-phylogenetic linear discriminant analysis (LDA) and the phylogenetic flexible discriminant analysis (pFDA) of Schmitz and Motani [75]. While LDA was applied to all extinct taxa and ootaxa examined, pFDA could only be used for fossil eggs of known taxonomic affinities (i.e., titanosaurs, *Lourinhanosaurus*, oviraptorosaurs, *Troodon*, and moas) as knowledge of the precise phylogenetic relationships between taxa is required for this method. Linear discriminant analysis was implemented with IBM SPSS Statistics v. 22.0.0, whereas pFDA was conducted in R3.1.3 with the phylo.fda.v0.2.R script provided by L. Schmitz (https://github.com/lschmitz/phylo.fda). LDA and pFDA were used to compare log-transformed values of eggshell porosity and egg mass of extinct archosaurs to those of living archosaurs (grouped *a priori* into open and covered nests categories) to infer the nest type for each extinct taxon/ootaxon. In order to test if a phylogenetic bias affects the form-function relationship in the dataset, the pFDA method provides an estimate of Pagel's lambda, where a lambda value of zero reveals no phylogenetic bias and a value of one indicates a strong bias where character evolution follows the Brownian motion model [74]. The pFDA method also provides a series of predictions for each taxon as a function of changing lambda value from 0 to 1. Prior probabilities of nest types, which are required for discriminant analyses, were based on the proportions of open and covered nest types found in the dataset of living archosaurs since the proportion of each nest type in extinct archosaurs is unknown. The misclassification rate was calculated for both LDA and pFDA based on the proportion of erroneously classified species. Since the misclassification rate of pFDA varies as a function of lambda values, the change in the overall misclassification rates through lambda values from 0 to 1 was also determined.

For pFDA, six extinct taxa were included in the composite phylogenetic tree of living archosaurs (S4 Fig). Because pFDA requires divergence times for estimation of branch length, phylogenetic relationships and divergence times of the extinct archosaur taxa were obtained from Bunce et al. [86], Choiniere et al. [87], Nesbitt [88], and Phillips et al. [89]. Terminal taxon ages were not precisely known for most extinct taxa but were approximated from fossil occurrence ages or geologic ages of formations in which taxa/ootaxa occur as reported by Gill [90], Rigby et al. [91], Chiappe et al. [49], Cunha et al. [92] and Varricchio et al. [17].

## Results

### Estimated D statistic

The estimated D statistic of nest type in the dataset of living archosaur species is -1.09, which is significantly different from 1 (*p* << 0.01) but not from 0 (*p* = 1.00). This D statistic indicates the presence of a strong phylogenetic bias in the distribution of nest types among archosaurs.

### Analysis of covariance (ANCOVA)

**Fig 2:**
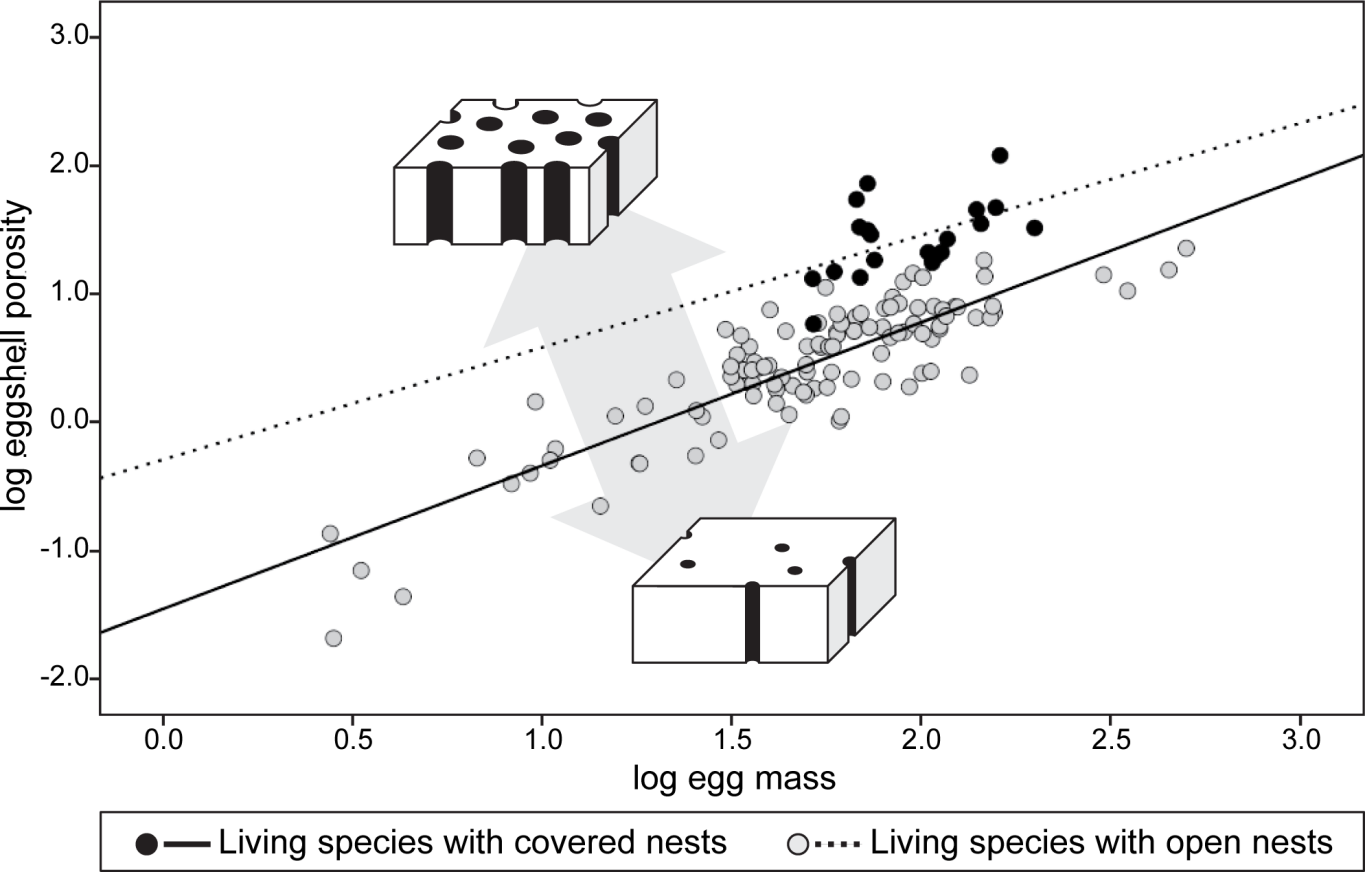
Bivariate plot of eggshell porosity and egg mass between living covered and open nesters. Eggshell porosity relative to egg mass is highly correlated to nest types (*p* < 0.01), as reflected by the different regression models between closed and open nesters.

Eggshell porosity, relative to egg mass, was compared among living archosaurs with covered (n = 20) and open nest types (n = 107) (Fig 2). Eggshell porosity between the two types is normally distributed (*p* = 0.49 and 0.15 for open and covered nest types, respectively) and homogeneity of variances is observed (*p* = 0.83), indicating that a parametric test is appropriate for the dataset. Eggshell porosity is shown to be strongly correlated to egg mass in taxa with open nests (r = 0.87, *p* < 0.01) and moderately correlated in species with covered nests (r = 0.52, *p* < 0.05) (Table 4). Both conventional and phylogenetically-corrected ANCOVA reveal that the slopes between these two nest types are not significantly different (*p* >> 0.05). Furthermore, the intercept of the regressions, and thus eggshell porosity relative to egg mass, is found to be significantly higher in the covered nest type than the open nest type (*p* < 0.01, Table 5; Fig 2) except using the PGLS model where branch length was estimated from divergence time (*p* = 0.11), which showed no significant difference in intercept. Of all the conventional and phylogenetically-corrected methods used, the RegOU model, where divergence time was used for branch length assignment, has the lowest AIC value and is thus considered the best-fit regression model tested (Table 5). The AIC value of the PGLS model is much higher than for the other models (i.e., OLS and RegOU) regardless of the methods for branch length assignment, indicating that the PGLS models were the poorer fit for our dataset.

**Table 4:** Results of conventional OLS regression models for living archosaur species.

Type	n	Slope	CI of slope	Intercept	CI of intercept	r^2^
Covered nester	20	0.874	0.161 to 1.587	-0.290	-1.699 to 1.119	0.269
Open nester	107	1.117	0.994 to 1.239	-1.453	-1.668 to -1.239	0.756

Abbreviations: CI, 95% confidence interval; n, sample size; r^2^, coefficient of determination.

**Table 5:** Results of conventional and phylogenetically-corrected ANCOVA for living archosaur species.

Branch length assignment	Model	F	d.f.	p	AIC
None	OLS	108.797	1, 124	<< 0.01	25.580
Branch length = 1	PGLS	11.568	1, 124	0.001	53.669
Branch length = 1	RegOU	70.111	1, 124	<< 0.01	25.007
Divergence time	PGLS	2.542	1, 124	0.113	72.841
Divergence time	RegOU	81.941	1, 124	<< 0.01	**17.578**

Abbreviations: AIC, akaike information criterion; d.f., degree of freedom; F, test statistic; OLS, ordinary least-squares; PGLS, phylogenetic generalized least-squares assumed Brownian motion process; RegOU, phylogenetic regression with Ornstein-Uhlenbeck process. The lowest AIC value is shown with bold.

### Discriminant analysis

**Fig 3:**
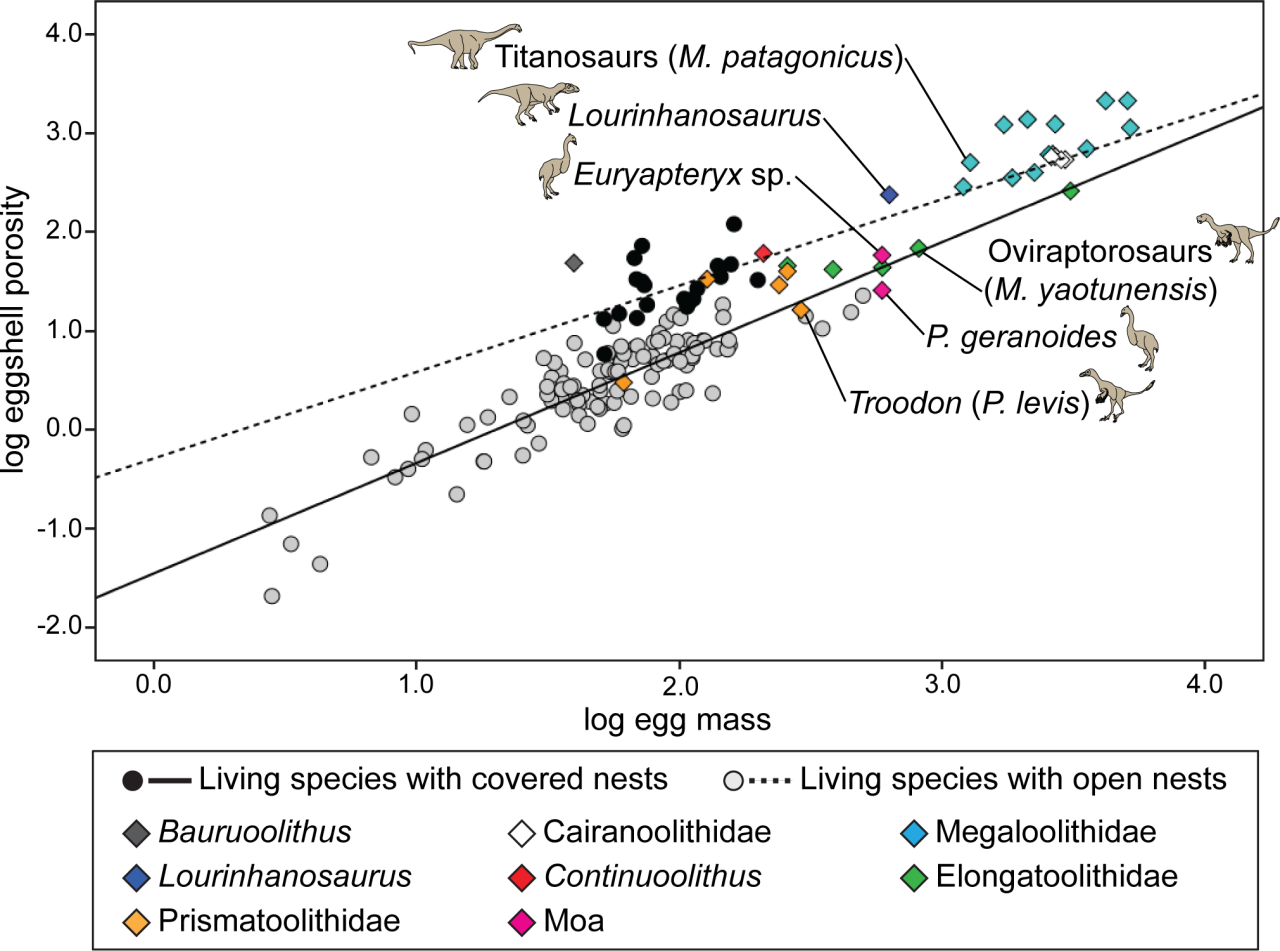
Bivariate plot of eggshell porosity and egg mass in both living and extinct archosaur taxa/ootaxa. Titanosaurs and *Lourinhanosaurus* show high eggshell porosity, comparable to living species with covered nests. In contrast, oviraptorosaurs, *Troodon*, and moas show lower eggshell porosity, similar to species with open nests.

When phylogenetic relationships are not taken into consideration, the linear discriminant analysis reveals that nest type can be predicted from eggshell porosity and egg mass among living archosaurs. From the dataset, 123 of the 127 extant bird and crocodylian species were classified correctly, resulting in an overall misclassification rate of only 3.15% (one open nester and three covered nesters were misclassified; Table 6). Applying this method to extinct archosaurs, crocodylomorphs (*Bauruoolithus*), possible ornithischians (Cairanoolithidae), sauropods (Megaloolithidae), and two non-avian theropods (*Lourinhanosaurus* and *Continuoolithus*) were classified as covered nesters, whereas most oviraptorosaurs (Elongatoolithidae), *Troodon* (i.e., *Prismatoolithus levis*), and other avian and non-avian theropods (most Prismatoolithidae and moas) were classified as open nesters (Table 7; Fig 3). Unlike other elongatoolithid and prismatoolithid eggs, *Elongatoolithus elongatus* and *Protoceratopsidovum minimum* were classified into the covered nest type. Posterior probabilities of extinct taxa/ootaxa were generally high (> 0.70), indicating that their predicted nest types were well differentiated from the other types. Two ootaxa, *E*. *elongatus* and *Pro*. *fluxuosum*, have posterior probabilities close to 0.50, which indicates that their eggshell porosity is close to the threshold between covered and open nest types.

**Table 6:** Cross-classification/ confusion matrix from LDA and pFDA.

	LDA		pFDA	
	Covered nest	Open nest	Covered nest	Open nest
Covered nest	17	1	0	0
Open nest	3	106	20	107
% Correct	85.000%	99.065%	0%	100%
Overall misclassification rate	3.150%		15.748%	

The true classifications are along the top and the predicted classifications are on the left-hand side.

**Table 7:** Inferred nest types for extinct archosaurs based on the linear discriminant analysis.

Family/oofamily	Taxon/ootaxon	Prediction	Posterior probabilities	
			Covered nest	Open nest
Krokolithidae	*Bauruoolithus fragilis*	Covered nest	1.000	0.000
Cairanoolithidae/ Fusioolithidae	*Cairanoolithus dughii*	Covered nest	0.747	0.253
	*Cairanoolithus roussetensis*	Covered nest	0.849	0.151
Megaloolithidae/ Fusioolithidae	*Megaloolithus aureliensis*	Covered nest	0.992	0.008
	*Megaloolithus mammilare*	Covered nest	0.881	0.119
	*Megaloolithus microtuberculata*	Covered nest	0.704	0.296
	*Megaloolithus patagonicus* (titanosaur sauropod)	Covered nest	0.985	0.015
	*Megaloolithus petralta*	Covered nest	0.889	0.111
	*Megaloolithus pseudomamillare*	Covered nest	0.804	0.196
	*Megaloolithus siruguei*	Covered nest	0.996	0.004
	*Megaloolithus* cf. *siruguei*	Covered nest	0.999	0.001
	*Megaloolithus* sp. (recrystallized)	Covered nest	0.753	0.247
	*Megaloolithus* sp. (non-recrystallized)	Covered nest	0.994	0.006
	Undetermined megaloolithid oospecies 1	Covered nest	0.896	0.104
	Undetermined megaloolithid oospecies 2	Covered nest	0.999	0.001
Oofamily Indet.	*Continuoolithus canadensis*	Covered nest	0.909	0.091
Allosauroidea?/ Coelurosauria?	*Lourinhanosaurus antunesi*	Covered nest	0.978	0.022
Elongatoolithidae	*Elongatoolithus andrewsi*	Open nest	0.139	0.861
	*Elongatoolithus elongatus*	Covered nest	0.543	0.457
	*Macroelongatoolithus xixiaensi*	Open nest	0.091	0.909
	*Macroolithus rugustus*	Open nest	0.033	0.967
	*Macroolithus yaotunensis* (oviraptorosaurs)	Open nest	0.061	0.939
Prismatoolithidae	*Prismatoolithus levis* (*Troodon formosus*)	Open nest	0.008	0.992
	*Protoceratopsidovum fluxuosum*	Open nest	0.402	0.598
	*Protoceratopsidovum minimum*	Covered nest	0.842	0.158
	*Protoceratopsidovum sincerum*	Open nest	0.185	0.815
	*Sankofa pyrenaica*	Open nest	0.003	0.997
Dinornithidae	*Euryapteryx* sp.	Open nest	0.107	0.893
	*Pachyornis geranoides*	Open nest	0.003	0.997

**Fig 4:**
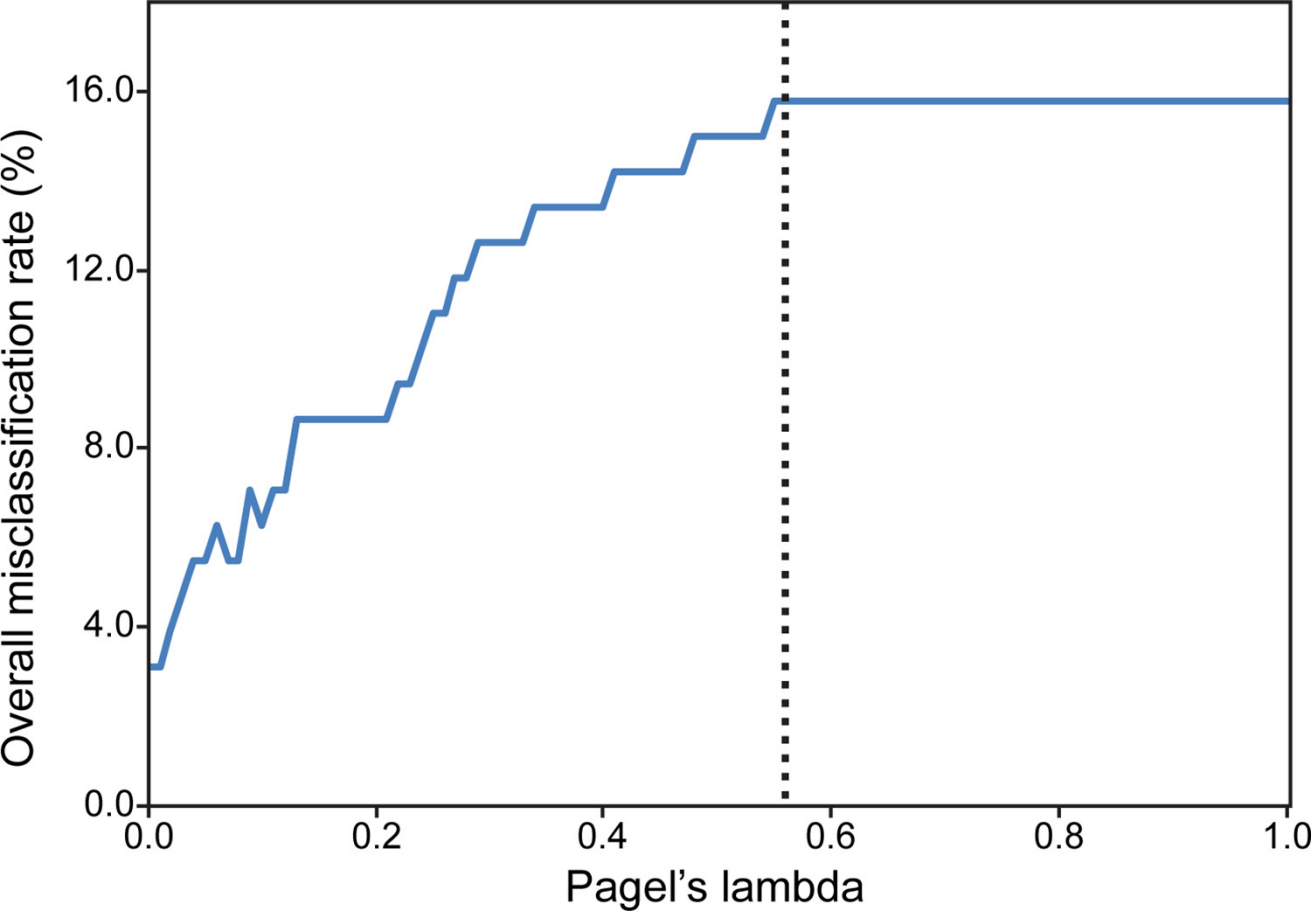
Misclassification rate of pFDA for living species through changing Pagel's lambda values. Dash line shows the optimal lambda value of 0.56. Note that the overall misclassification rate increases with increasing lambda values from 0 to 1.

**Fig 5:**
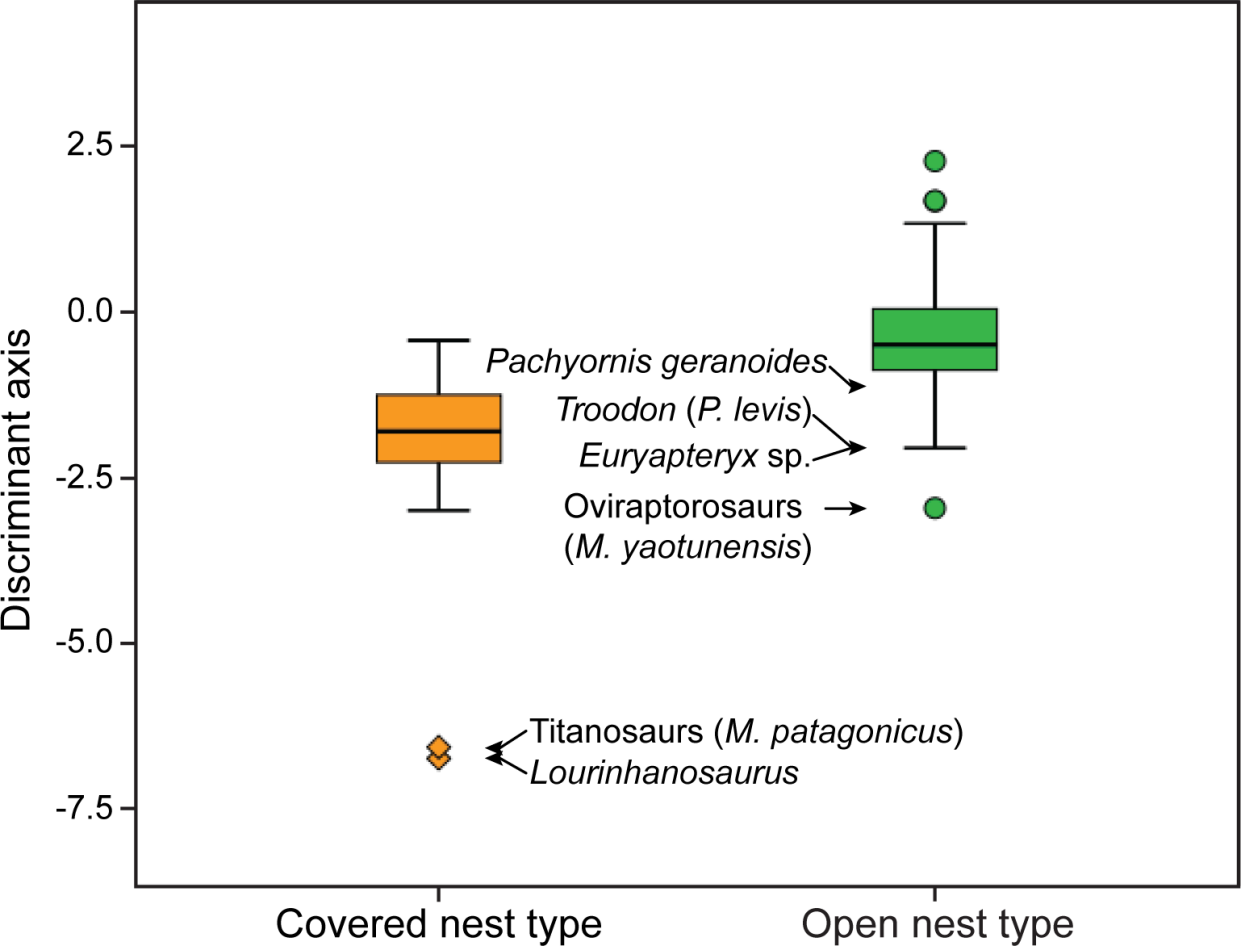
Comparison of the discriminant function between covered and open nesters in living and fossil archosaurs. Horizontal bars inside boxes represent medians, lower and upper ends of boxes are the 25% and 75% quartiles, respectively, and whiskers represent the smallest and largest cases. Outliers are represented by dots and extremes by diamonds. Note that covered nesters show relatively lower values than open nesters.

**Fig 6:**
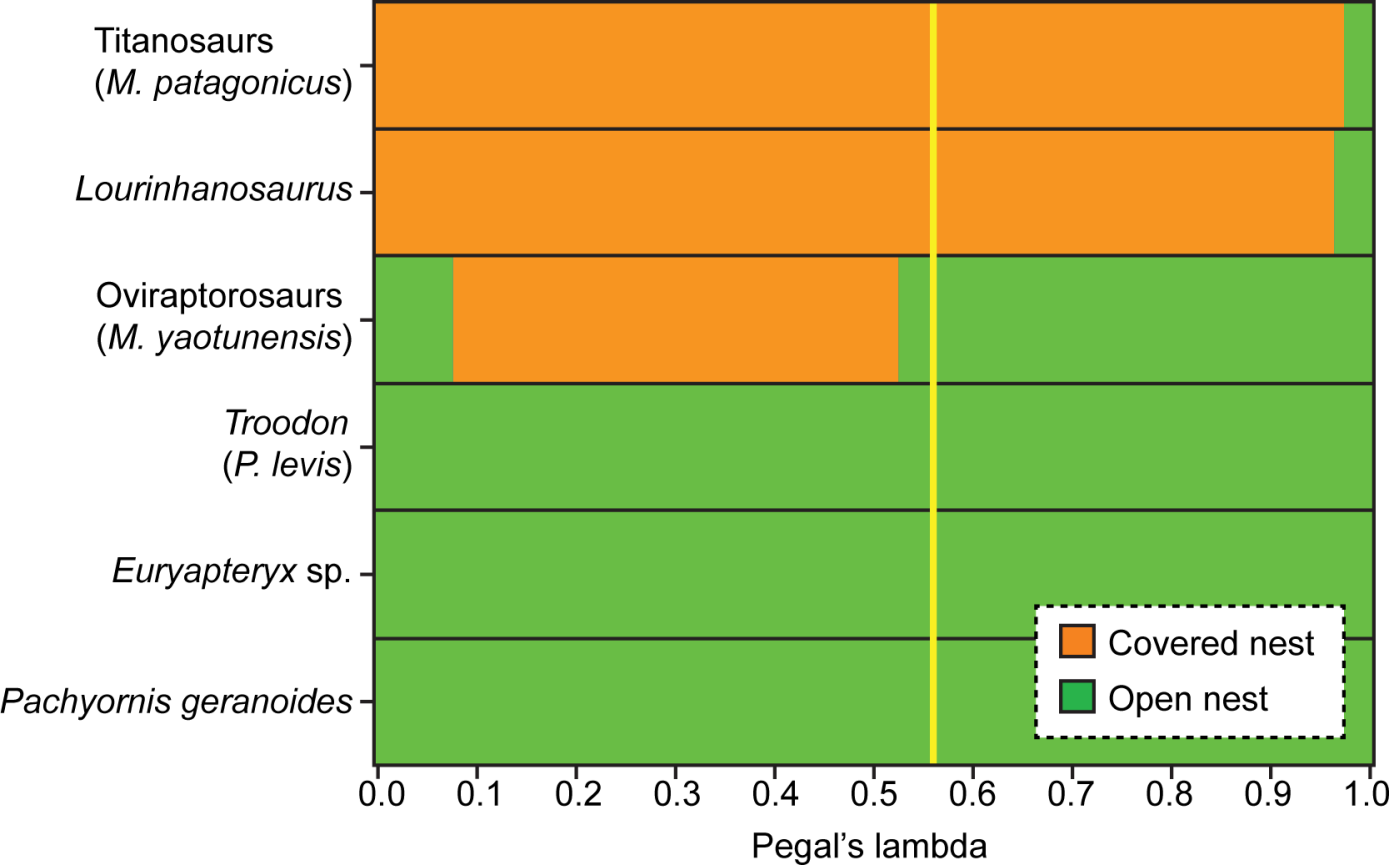
Inferred nest type for six extinct archosaurs as a function of Pagel's lambda values. Inferred nest type is generally consistent across all lambda values, except for oviraptorosaurs where inferred nest type changes when the lambda value varies between 0.08 and 0.52, and for titanosaurs and *Lourinhanosaurus*, which change to open nesters when lambda values approach one. The yellow line indicates the optimal lambda value (0.56).

When phylogenetically-corrected methods are used, the pFDA reveals that the optimum Pagel's lambda value is 0.56, which indicates that a moderately high phylogenetic bias exists in the dataset. The pFDA correctly classified 107 of 127 living species, resulting in an overall misclassification rate of 15.75% (Table 6). Although all extant archosaurs with an open nest type were classified correctly, none of the 20 species with a covered nest type was classified correctly. Thus, the pFDA misclassification rate for extant covered nesters is 100%. The overall misclassification rate increases with increasing Pagal's lambda value, where the lowest misclassification rate was found at lambda values ≤0.01 (Fig 4). At optimal Pagel's lambda value, results of the phylogenetically-corrected discriminant analysis are consistent with the results of the conventional method (i.e., titanosaurs and *Lourinhanosaurus* are covered nesters, and oviraptorosaurs, *Troodon*, and moas are open nesters; Table 8, Fig 5). Results of the pFDA do not change at non-optimal Pagel's lambda values except for oviraptorosaurs, which change to covered nesters at lambda values between 0.08 and 0.52, and for titanosaurs and *Lourinhanosaurus*, which change to open nesters when lambda values approach one (Fig 6).

**Table 8:** Inferred dinosaur nest types based on the phylogenetic flexible discriminant analysis.

Taxon/ootaxon	Prediction	Posterior probabilities	
		Covered nest	Open nest
*Megaloolithus patagonicus* (titanosaur sauropod)	Covered nest	0.888	0.112
*Lourinhanosaurus antunesi*	Covered nest	0.898	0.102
*Macroolithus yaotunensis* (oviraptorosaurs)	Open nest	0.487	0.513
*Prismatoolithus levis* (*Troodon formosus*)	Open nest	0.357	0.643
*Euryapteryx* sp.	Open nest	0.358	0.642
*Pachyornis geranoides*	Open nest	0.243	0.757

### Interpretation of Statistical Results

Conventional and phylogenetically-corrected discriminant analyses produce results that differ for living taxa (i.e., higher misclassification rate in phylogenetically-corrected analysis, particularly among covered nesters) but that are consistent for fossil taxa (i.e., extinct taxa are assigned to the same nest type in both types of analysis). The LDA reveals that eggshell porosity (relative to egg mass) predicts accurately nest type in living archosaurs (i.e., low misclassification rate). Since the precise taxonomic affinity, and hence phylogenetic position, is unknown for most fossil eggs, conventional methods such as LDA should be used for these specimens in order to obtain reliable inferences of nesting habits. In contrast when phylogenetic relationships are taken into consideration, misclassification rate is high among living species due to the presence of a phylogenetic bias in the dataset (i.e., high optimal Pagal’s lambda value in pFDA and low D statistic value). This phylogenetic bias is due to the fact that covered nests are restricted to two relatively basal clades of extant archosaurs (i.e., Crocodylia and Megapodiidae), resulting in a clumped phylogenetic distribution of this trait (S3 Fig). Previous studies that have used the pFDA method usually obtained lower optimal lambda values (< 0.20; see [75,93–95]), which indicates that the phylogenetic distribution of their traits was more randomly distributed (i.e., less clumped) than in our study.

When phylogenetic relationships were taken into consideration with the pFDA method, the misclassification rates increased. The overall misclassification rate increases with increasing lambda values (Fig 4): the misclassification rate is at its lowest (3.15%) at low Pagal’s lambda values (≤0.01) and at its highest (15.75%) at high lambda values (≥ 0.55), including at the optimal lambda value. This pattern is opposite that observed by Motani and Schmitz [74], the only study to have shown a change in misclassification rate with changing lambda values, where they observed the lowest misclassification rate around the optimal Pagal’s lambda value. The fact that pFDA assumes the Brownian motion model, which was the worst fit model for our dataset according to pcANCOVA, could explain the poor performance of this method in our study. Other methods, such as the Ornstein-Uhlenbeck model, which produced the best fit in pcANCOVA, may work better for our dataset, although this model cannot be performed in pFDA. A possible solution to this problem is to use LDA rather than pFDA until the latter method is developed further. Regardless of the high misclassification rate of the pFDA, inferences of nest type for extinct archosaurs are consistent between LDA and pFDA (at optimal lambda value).

## Discussion

Our results reveal that eggshell porosity, expressed relative to egg mass, is highly correlated with nest type among living archosaurs in that eggs incubated in covered nests have a significantly higher eggshell porosity than those incubated in open nests. This newly discovered correlation permits the use of a discriminant analysis (LDA, pFDA) to infer nest type among extinct archosaurs, which could not be achieved with previous methods. Although pFDA struggled to correctly classify living species based on eggshell porosity, possibly due to built-in assumptions of evolutionary mode, results of LDA and pFDA were consistent for fossil taxa.

The eggshell porosity approach developed here is methodologically consistent and uses statistical rigor to infer nest type in extinct archosaurs, unlike the previous method based on water vapor conductance (G_H2O_). For the G_H2O_ method, nest type inference relies on comparisons between G_H2O_ values measured experimentally for fresh eggs for living taxa and G_H2O_ values estimated from egg/eggshell morphometric data for fossil taxa. The issues with this method are that: 1) the experimental and morphometric approaches do not produce results that are mutually consistent for living species, and thus comparisons between them should be avoided [18], and 2) a correlation between G_H2O_ (neither experimental nor morphometric) and nest type has never been established in living taxa. In contrast, the eggshell porosity method proposed here relies exclusively on egg/eggshell morphometric data obtained from both living and extinct taxa and is based on a demonstrated (and statistically-significant) correlation between eggshell porosity and nest type.

Our study reveals that sauropods, the theropod *Lourinhanosaurus*, and the potential ornithischian ootaxon *Cairanoolithus* had covered nests based on relatively high eggshell porosity, a result that is in agreement with most previous G_H2O_ studies [14,15,35,40], except one [16]. Most oviraptorosaurs (Elongatoolithidae) are classified here as open nesters due to relatively low porosity values (also determined by [44]), although several previous G_H2O_ studies have inferred covered nests (e.g., [15,41,42,96]). This discrepancy may be due to the fact that nest type was determined subjectively in these earlier studies, due to a lack of rigorous statistical analysis (Fig 3). *Troodon formosus* and two of three *Protoceratopsidovum* oospecies have relatively low eggshell porosity values and are inferred to have been open nesters, results consistent with the previously reported low G_H2O_ values for *Troodon* and *Protoceratopsidovum* [15,17,31].

**Fig 7:**
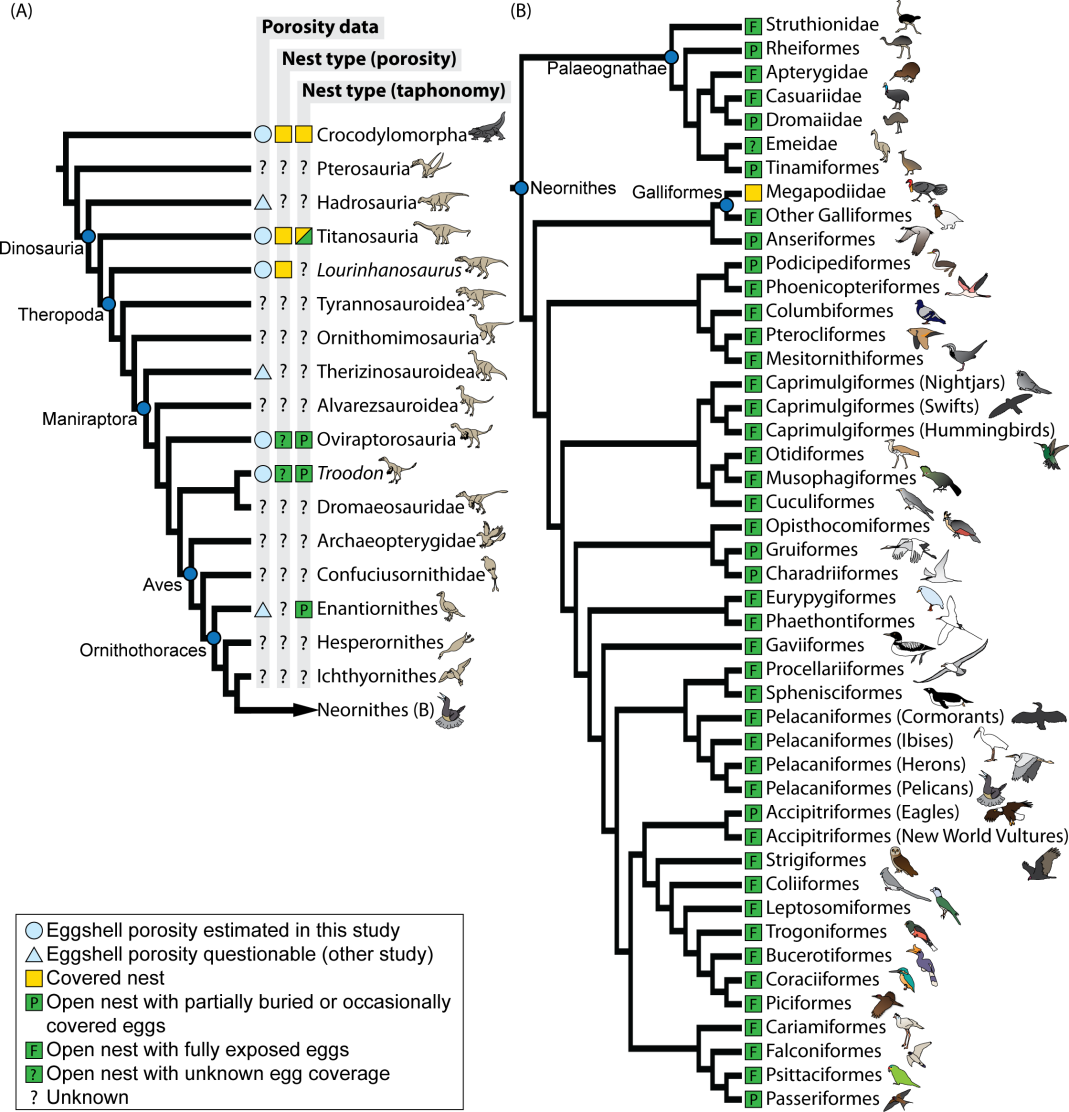
Evolution of nest types among archosaurs. (A) Phylogeny of archosaurs with inferred nest types based on eggshell porosity and taphonomic evidence. Covered nests are the primitive condition for dinosaurs; open nests and brooding behavior were present among non-avian maniraptoran theropods but may have first appeared earlier. Although the eggs of early open nesters were still partially covered by substrate, open nests with fully exposed eggs likely arose among Euornithes. (B) Phylogeny of Neornithes with inferred nest types based on eggshell porosity (Emeidae) and literature (other birds). Open nests with fully exposed eggs are the primitive condition for modern birds, although secondary reversal to partial egg burial occurred independently in several clades. Information for bird orders which include species that partially bury the eggs (Charadriiformes) or occasionally cover the eggs in open nests (Accipitriformes, Anseriformes, Charadriiformes, Gruiformes, Passeriformes, Podicipediformes, Struthioniformes, Tinamiformes) was taken from [97–103]. Cladograms are based on [73,88,104–106].

When considered in a phylogenetic context, our results shed light on the evolution of nest types among dinosaurs (Fig 7). The presence of covered nests in crocodylomorphs, titanosaurs, the theropod *Lourinhanosaurus*, and probably ornithischians (*Cairanoolithus*) indicates that these nests were likely the primitive condition in Dinosauria and possibly Archosauria. In contrast, open nests with partly or fully exposed eggs were present among oviraptorosaurs, troodontids, and birds, and thus were probably also present in the last common ancestor of oviraptorosaurs and troodontids (i.e., a non-avian maniraptoran). Open nests may have appeared even earlier in theropod evolution but a large phylogenetic gap in the fossil record of their eggs precludes a more precise determination (Fig 7). Nevertheless, our results reveal that open nests first appeared in non-avian theropods well before the origin of Aves.

The evolutionary transition in nest types observed among non-avian theropods may also be linked to changes in other nesting habits, such as brooding behavior and the arrangement of eggs in the nest. A plausible scenario is that open nests and brooding behaviors evolved in association because the transfer of body heat to the eggs would be effective only if the eggs were exposed (at least partially) in the nest so as to be in contact with the parent (e.g., [5]). This hypothesis is supported by the discovery of both oviraptorosaur and troodontid skeletons sitting atop or in contact with the eggs [5,59,60,64,107], which suggests that these early open nesters could have been brooders. Primitively, eggs laid in open nests may have been partially buried in substrate or nesting material, as suggested by the taphonomy of troodontid and enantiornithine bird clutches [8,108,109] and the multi-layered arrangement of oviraptorosaur clutches [44]. It is only later in avian evolution, presumably among euornithine birds, that eggs were left fully exposed in open nests, a condition observed in most extant brooding birds. Some neornithine taxa (e.g., waders, grebes, some waterfowl, screamers, and tinamous), however, likely secondarily evolved behaviors to partially bury their eggs during incubation [101,110], for either thermoregulation or concealment purposes ([101,111]; Fig 7).

The evolution of open nests and brooding behavior may have played a key role in allowing maniraptoran theropods, including birds, to exploit a greater diversity of locations for nesting. Nest location for covered nesters (i.e., crocodylians and megapodes) is restricted to the ground because heat and humidity is required from the nesting materials/substrate for incubation [4]. Conversely, reliance on body heat for egg incubation in fully open nesters probably freed maniraptorans to exploit new environments to build their nests (e.g., trees, cliffs, caves). Furthermore, this greater nesting freedom may have lessened the odds of nesting failure due to predation, flooding, or torrential rainfall, factors commonly adversely affecting the hatching success of covered nests on the ground (e.g., [4,112–114], and consequently may have played a role in the evolutionary success and adaptive radiation of maniraptorans [115].

## Supporting Information

S1 Text Use of eggshell porosity as an indicator of nest types in archosaurs. (DOCX)

S1 Fig Cladogram of 196 living bird species for comparisons of M_H2O_ (phylogenetic generalized least square model in S2 Fig). (DOCX)

S2 Fig Bivariate plot of daily loss of water vapor and egg mass in living species. (DOCX)

S3 Fig Cladogram of 127 living archosaur species for pcANCOVA. (DOCX)

S4 Fig Cladogram of 133 living and extinct archosaur taxa for pFDA. (DOCX)

S1 Table Daily loss of water vapor (M_H2O_) and egg mass (M) in 196 living archosaur species. (DOCX)

S2 Table Museum specimens assessed for eggshell porosity in this study. (DOCX)

S3 Table Eggshell porosity (A_p_∙Ls-1) and egg mass (M) of living and extinct archosaurs. (DOCX)

S4 Table Nest type classification for living archosaur species. (DOCX)
