# Supplementary material for: Eggshell Porosity Provides Insight on Evolution of Nesting in Dinosaurs
Source: PLoS One. 2015 Nov 25;10(11):e0142829. doi: 10.1371/journal.pone.0142829 (PMC4659668; doi:10.1371/journal.pone.0142829)
Supplement: S1 Text — (DOCX) [file pone.0142829.s001.docx]

**S1 Text. Use of eggshell porosity as an indicator of nest types in archosaurs.**

It can be hypothesized that values of eggshell porosity (A_p_∙L_s_^-1^) differ between species with covered and open nests in living archosaurs. Because pore canals are the only pathways for water vapor in eggshell, water vapor conductance (G_H2O_) can be calculated using eggshell properties (morphometric method), which is deduced from Fick's first law of gas diffusion:

[S1-1] $G_{mo}=\frac{{c\cdot D}_{H2O}}{R\cdot T}\cdot\frac{A_{p}}{L_{s}}$

where c is a unit conversion constant (mgH_2_O∙sec∙day^-1^∙mol^-1^), D_H2O_ is the diffusion coefficient of water vapor (mm^2^∙sec^-1^), G_mo_ is the morphometrically-derived G_H2O_ (mgH_2_O∙day^-1^∙torr^-1^), R is the universal gas constant (mm^3^∙torr∙mol^-1^∙K^-1^), and T is the absolute temperature of incubation (°K) [1]. Because D_H2O_, T, c, and R are assumed to be consistent among species (e.g., [1-3]), Equation S1-1 can be simplified [3]:

[S1-2] $G_{mo}=2.1\cdot\frac{A_{p}}{L_{s}}$

Also, G_H2O_ is experimentally estimated (experimental method) using fresh eggs as:

[S1-3] $G_{ex}=\frac{M_{H2O}}{P_{egg}-P_{nest}}$

where M_H2O_ is the daily loss of water vapor (mgH_2_O∙day^-1^), G_ex_ is the experimentally-derived G_H2O_ (mgH_2_O∙day^-1^∙torr^-1^), P_egg_ is water vapor pressure inside of egg (torr), and P_nest_ is water vapor pressure of nest (torr) [1]. Although log G_mo_ may not be equivalent to log G_ex_ due to potential systematic errors between the morphometric and experimental methods, these variables show a significant positive correlation [3]. Thus,

[S1-4] ${\log G}_{mo}\propto{\log G}_{ex}$

and

[S1-5] $\log\left( \frac{A_{p}}{L_{s}} \right)\propto\log\left( \frac{M_{H2O}}{P_{egg}-P_{nest}} \right)$

Thus, log A_p_∙L_s_^-1^ is proportional to log M_H2O_ and inversely proportional to log (P_egg_ - P_nest_). While P_egg_ is assumed to be consistent among species (40 to 50 torr in birds [4,5] and approximately 32 torr in crocodilians [6]), P_nest_ is significantly higher in species with covered nests (mean 31.74 torr) than in those with open nests (mean 20.68 torr) [6]. Thus, log (P_egg_ - P_nest_) should be lower in species with covered nests, especially crocodilians, than in those with open nests. Also, log M_H2O_ values, relative to log egg mass (M, in g), are consistent among species, regardless of nest types (Table S1; Figure S2), because no outlier was detected based on a Generalized Extreme Studentized Deviate test using MedCalc Statistical Software v. 13.0 (MedCalc Software bvba, Ostend, Belgium; http://www.medcalc.org; 2014). Therefore, it can be assumed that A_p_∙L_s_^-1^ is primarily affected by P_nest_ and that log A_p_∙L_s_^-1^ is expected to be higher in species that build covered nests (i.e., high P_nest_) than in those that build open nests (i.e., lower P_nest_).

**References**

1. Ar A, Paganell CV, Reeves RB, Greene DG, Rahn H (1974) Avian egg: water vapor conductance, shell thickness, and functional pore area. Condor 76: 153-158.

2. Ar A, Rahn H (1985) Pores in avian eggshells: gas conductance, gas exchange and embryonic growth rate. Respiration Physiology 61: 1-20.

3. Tanaka K, Zelenitsky DK (2014) Comparisons between experimental and morphometric water vapor conductance in the eggs of extant birds and crocodiles: implications for predicting nest type in dinosaurs. Canadian Journal of Zoology 92: 1049-1058.

4. Rahn H (1984) Factors controlling the rate of incubation water loss in bird eggs. In: Seymour RS, editor. Respiration and metabolism of embryonic vertebrates. Dordrecht, Netherlands: Dr. W. Junk Publications. pp. 271-288.

5. Booth DT, Thompson MB (1991) A comparison of reptilian eggs with those of megapode birds. In: Deeming DC, Ferguson MWJ, editors. Egg Incubation: Its Effects on Embryonic Development in Birds and Reptiles. Cambridge, United Kingdom: Cambridge University Press. pp. 325-344.

6. Tanaka K, Zelenitsky DK (2014) Relationships between nest humidity and nest types in living archosaurs. Historical Biology 26: 122-131.
