# Supplementary material for: Eggshell Porosity Provides Insight on Evolution of Nesting in Dinosaurs
Source: PLoS One. 2015 Nov 25;10(11):e0142829. doi: 10.1371/journal.pone.0142829 (PMC4659668; doi:10.1371/journal.pone.0142829)
Supplement: S1 Fig — (DOCX) [file pone.0142829.s002.docx]

**S1 Fig.** Cladogram of 196 living bird species for comparisons of M_H2O_ (phylogenetic generalized least square model in S2 Fig.). Modified from [1-30]**.**


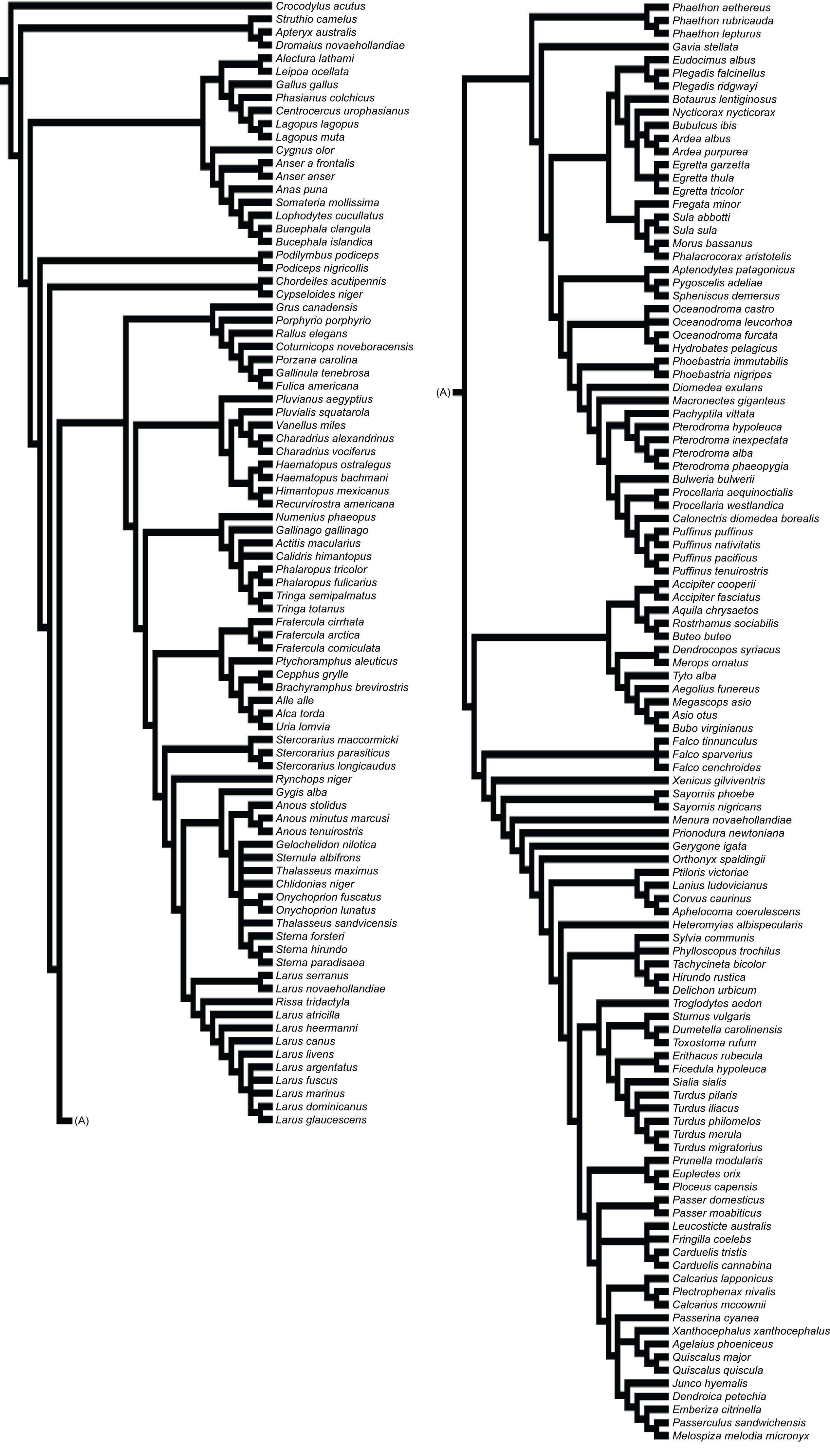


**References**

1. Livezey BC (1996) A phylogenetic analysis of modern pochards (Anatidae: Aythyini). Auk 113: 74-93.

2. Sheldon FH (1987) Phylogeny of herons estimated from DNA-DNA hybridization data. Auk 104: 97-108.

3. Livezey BC (1995) Phylogeny and evolutionary ecology of modern seaducks (Anatidae, Mergini). Condor 97: 233-255.

4. Livezey BC (2001) A phylogenetic analysis of modern sheldgeese and shelducks (Anatidae, Tadornini). Ibis 139: 51-66.

5. Sheldon FH, Slikas B (1997) Advances in ciconiiform systematics 1976-1996. Colonial Waterbirds 20: 106-114.

6. Kennedy M, Spencer HG (2000) Phylogeny, biogeography, and taxonomy of Australasian Teals. Auk 117: 154-163.

7. Kennedy M, Spencer HG (2004) Phylogenies of the Frigatebirds (Fregatidae) and Tropicbirds (Phaethonidae), two divergent groups of the traditional order Pelecaniformes, inferred from mitochondrial DNA sequences. Molecular Phylogenetics and Evolution 31: 31-38.

8. Sheldon FH, Jones CE, McCracken KG (2000) Relative patterns and rates of evolution in heron nuclear and mitochondrial DNA. Molecular Biology and Evolution 17: 437-450.

9. Wink M, Heidrich P (2000) Molecular systematics of owls (Strigiformes) based on DNA-sequences of the mitochondrial cytochrome b gene. In: Chancellor RD, Meyburg B-U, editors. Raptors at Risk. London: WWGBP, Hancock House. pp. 819–828.

10. Johnson KP, De Kort S, Dinwoodey K, Mateman AC, Ten Cate C, Lesselles CM, et al. (2001) A molecular phylogeny of the dove genera *Streptopella* and *Columba*. Auk 118: 874-887.

11. Kennedy M, Page RDM (2002) Seabird supertrees: combining partial estimates of procellariiform phylogeny. Auk 119: 88-108.

12. de Kloet RS, de Kloet SR (2005) The evolution of the spindlin gene in birds: sequence analysis of an intron of the spindlin W and Z gene reveals four major divisions of the Psittaciformes. Molecular Phylogenetics and Evolution 36: 706-721.

13. Fjeldsa J (2004) The Grebes: Podicipedidae. Oxford, United Kingdom: Oxford University Press. 264 p.

14. Krebs EA, Putland DA (2004) Chic chicks: the evolution of chick ornamentation in rails. Behavioral Ecology 15: 946-951.

15. Thomas GH, Wills MA, Szekely T (2004) A supertree approach to shorebird phylogeny. BMC Evolutionary Biology 4: 10.1186/1471-2148-1184-1128.

16. Bertelli S, Giannini NP (2005) A phylogeny of extant penguins (Aves: Sphenisciformes) combining morphology and mitochondrial sequences. Cladistics 21: 209-239.

17. Lerner HRL, Mindell DP (2005) Phylogeny of eagles, Old World vultures, and other Accipitridae based on nuclear and mitochondrial DNA. Molecular Phylogenetics and Evolution 37: 327-346.

18. Crowe TM, Bowie RCK, Bloomer P, Mandiwana TG, Hedderson TAJ, Randi E, et al. (2006) Phylogenetics, biogeography and classification of, and character evolution in, gamebirds (Aves: Galliformes): effects of character exclusion, data partitioning and missing data. Cladistics 22: 495-532.

19. Ericson PGP, Anderson CL, Britton T, Elzanowski A, Johansson US, Kellersjo M, et al. (2006) Diversification of Neoaves: integration of molecular sequence data and fossils. Biology Letters 2: 543-547.

20. Jonsson KA, Fjeldsa J (2006) A phylogenetic supertree of oscine passerine birds (Aves : Passeri). Zoologica Scripta 35: 149-186.

21. Ksepka DT, Bertelli S, Giannini NP (2006) The phylogeny of the living and fossil Sphenisciformes (penguins). Cladistics 22: 412-441.

22. Griffiths CS, Barrowclough GF, Groth JG, Mertz LA (2007) Phylogeny, diversity, and classification of the Accipitridae based on DNA sequences of the RAG-1 exon. Journal of Avian Biology 38: 587-602.

23. Proudfoot GA, Gehlbach FR, Honeycutt RL (2007) Mitochondrial DNA variation and phylogeography of the Eastern and Western screech-owls. Condor 109: 617-627.

24. Wright TF, Schirtzinger EE, Matsumoto T, Eberhard JR, Graves GR, Sanchez JJ, et al. (2008) A multilocus molecular phylogeny of the parrots (Psittaciformes): support for a Gondwanan origin during the Cretaceous. Molecular Biology and Evolution 25: 2141-2156.

25. Gonzalez J, Duttmann H, Wink M (2009) Phylogenetic relationships based on two mitochondrial genes and hybridization patterns in Anatidae. Journal of Zoology 279: 310-318.

26. Mayr G (2010) Parrot interrelationships: morphology and the new molecular phylogenies. Emu 110: 348-357.

27. McCracken KG, Barger CP, Sorenson MD (2010) Phylogenetic and structural analysis of the HbA (α^A^/β^A^) and HbD (α^D^/β^A^) hemoglobin genes in two high-altitude waterfowl from the Himalayas and the Andes: bar-headed goose (*Anser indicus*) and Andean goose (*Chloephaga melanoptera*). Molecular Phylogenetics and Evolution 56: 649-658.

28. Smith ND (2010) Phylogenetic analysis of Pelecaniformes (Aves) based on csteological data: implications for waterbird phylogeny and fossil calibration studies. Plos One 5: e13354 doi: 13310.11371/journal.pone.0013354.

29. Yang R, Wu XB, Yan P, Su X, Yang BH (2010) Complete mitochondrial genome of *Otis tarda* (Gruiformes: Otididae) and phylogeny of Gruiformes inferred from mitochondrial DNA sequences. Molecular Biology Reports 37: 3057-3066.

30. Jarvis ED, Mirarab S, Aberer AJ, Li B, Houde P, Li C, et al. (2014) Whole-genome analyses resolve early branches in the tree of life of modern birds. Science 346: 1320-1331.
