# Supplementary material for: Eggshell Porosity Provides Insight on Evolution of Nesting in Dinosaurs
Source: PLoS One. 2015 Nov 25;10(11):e0142829. doi: 10.1371/journal.pone.0142829 (PMC4659668; doi:10.1371/journal.pone.0142829)
Supplement: S2 Fig — (DOCX) [file pone.0142829.s003.docx]

**S2 Fig. Bivariate plot of daily loss of water vapor and egg mass in living species.** Ordinary least square (OLS) and phylogenetic generalized least square (PGLS) regressions as well as the 95% confidence intervals (CIs) were conducted for living archosaur species using IBM SPSS Statistics v. 22.0.0 (IBM SPSS Inc.) and PDAP module v.1.15 [1] of the software Mesquite 3.02 [2]. For the phylogenetic approach, a phylogenetic tree of 196 species was reconstructed (S1 Fig.) and tree lengths were assigned based on Pagel's method [3] and divergence time. White and black dots represent open and covered nesters, respectively. Note that log M_H2O_ is strongly correlated with log M (*r^2^* = 0.888 for OLS, 0.638 for PGLS with Pagel's branch length method, and 0.670 for PGLS with the divergence time method).


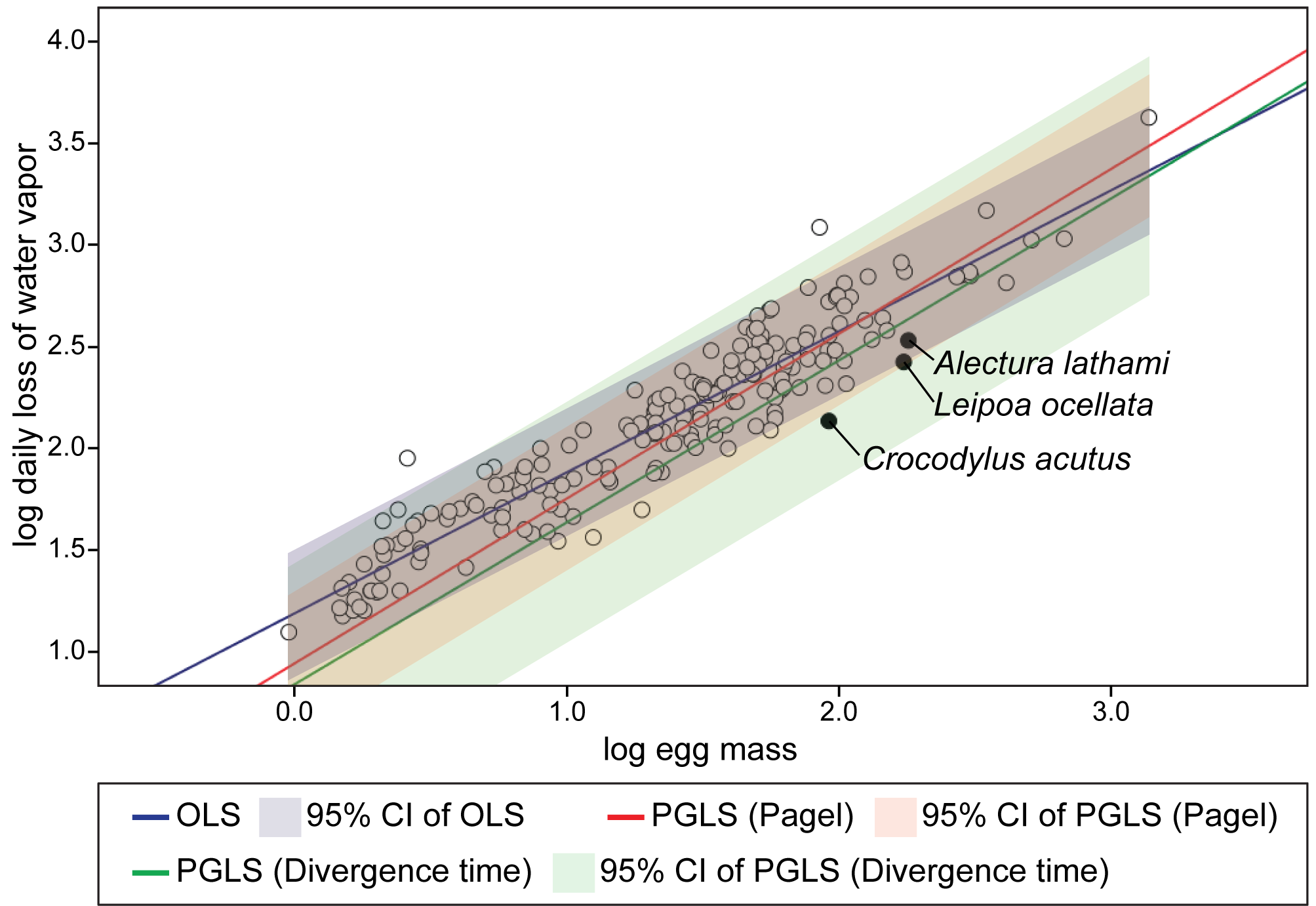


**References**

1. Midford PE, Garland Jr. T, Maddison WP (2010) PDAP package of Mesquite, version 1.15. See http://mesquiteproject.org/pdap_mesquite/index.html.

2. Maddison WP, Maddison DR (2010) Mesquite: a modular system for evolutionary analysis. Version 2.73. See http://mesquiteproject.org.

3. Pagel MD (1992) A method for the analysis of comparative data. Journal of Theoretical Biology 156: 431-442.
