# Supplementary material for: Eggshell Porosity Provides Insight on Evolution of Nesting in Dinosaurs
Source: PLoS One. 2015 Nov 25;10(11):e0142829. doi: 10.1371/journal.pone.0142829 (PMC4659668; doi:10.1371/journal.pone.0142829)
Supplement: S1 Table — (DOCX) [file pone.0142829.s006.docx]

**S1 Table. Daily loss of water vapor (M_H2O_) and egg mass (M) in** 196 living archosaur species.

Values of M and M_H2O_, both derived from natural incubation experiments, were compiled from the literature. Some M_H2O_ values were calculated in the context of this study based on values reported in the literature of mass loss of eggs over known time intervals. When multiple values of log M_H2O_ and log M were available for a species, mean values were calculated.

| Taxon | log M_H2O_ | log M | References |
| --- | --- | --- | --- |
| Accipitridae | | | |
| *Accipiter cooperii* | 2.328 | 1.462 | [1] |
| *Accipiter fasciatus* | 2.114 | 1.583 | [2] |
| *Aquila chrysaetos* | 2.642 | 2.161 | [1,3] |
| *Buteo buteo* | 2.398 | 1.797 | [4,5] |
| *Rostrhamus sociabilis* | 2.292 | 1.501 | [6] |
| Anseriformes | | | |
| *Anas puna* | 2.595 | 1.659 | [7] |
| *Anser a. frontalis* | 2.845 | 2.107 | [8,9] |
| *Anser anser* | 2.869 | 2.241 | [10] |
| *Bucephala clangula* | 2.428 | 1.806 | [11] |
| *Bucephala islandica* | 2.398 | 1.829 | [12] |
| *Cygnus olor* | 3.170 | 2.542 | [13] |
| *Lophodytes cucullatus* | 2.517 | 1.768 | [14] |
| *Somateria mollissima* | 2.812 | 2.020 | [5,15-17] |
| Apodiformes | | | |
| *Cypseloides niger* | 1.672 | 0.722 | [18] |
| Caprimulgiformes | | | |
| *Chordeiles acutipennis* | 1.598 | 0.760 | [19] |
| Charadriiformes | | | |
| *Actitis macularius* | 1.820 | 0.982 | [20] |
| *Alca torda* | 2.556 | 1.964 | [5,21] |
| *Alle alle* | 2.314 | 1.491 | [22,23] |
| *Anous minutus marcusi* | 2.025 | 1.375 | [24] |
| *Anous stolidus* | 2.102 | 1.548 | [24,25] |
| *Anous tenuirostris* | 2.025 | 1.394 | [26] |
| *Brachyramphus brevirostris* | 2.477 | 1.732 | [27] |
| *Calidris himantopus* | 2.461 | 1.683 | [28] |
| *Cepphus grylle* | 2.408 | 1.706 | [5,16,29] |
| *Charadrius alexandrinus* | 1.579 | 0.874 | [19] |
| *Charadrius vociferus* | 1.835 | 1.159 | [19,30,31] |
| *Chlidonias niger* | 1.851 | 1.026 | [32] |
| *Fratercula arctica* | 2.469 | 1.831 | [5,16] |
| *Fratercula cirrhata* | 2.482 | 1.959 | [33] |
| *Fratercula corniculata* | 2.534 | 1.878 | [34] |
| *Gallinago gallinago* | 2.114 | 1.220 | [31] |
| *Gelochelidon nilotica* | 2.068 | 1.455 | [35] |
| *Gygis alba* | 1.883 | 1.349 | [24,36] |
| *Haematopus bachmani* | 2.398 | 1.663 | [37] |
| *Haematopus ostralegus* | 2.505 | 1.638 | [38] |
| *Himantopus mexicanus* | 2.180 | 1.322 | [19] |
| *Larus argentatus* | 2.721 | 1.962 | [5,10,16,38,39] |
| *Larus atricilla* | 2.362 | 1.649 | [40] |
| *Larus canus* | 2.556 | 1.715 | [5,16,38] |
| *Larus dominicanus* | 3.087 | 1.929 | [41,42] |
| *Larus fuscus* | 2.568 | 1.885 | [38] |
| *Larus glaucescens* | 2.753 | 1.992 | [43,44] |
| *Larus heermanni* | 2.444 | 1.728 | [45] |
| *Larus livens* | 2.744 | 1.990 | [45] |
| *Larus marinus* | 2.744 | 2.042 | [5,10,16,39] |
| *Larus novaehollandiae* | 2.230 | 1.623 | [46] |
| *Larus serranus* | 2.679 | 1.744 | [47] |
| *Numenius phaeopus* | 2.575 | 1.688 | [48] |
| *Onychoprion fuscatus* | 2.279 | 1.553 | [24] |
| *Onychoprion lunatus* | 2.039 | 1.458 | [49,50] |
| *Phalaropus fulicarius* | 1.818 | 0.898 | [51] |
| *Phalaropus tricolor* | 1.701 | 0.978 | [31] |
| *Pluvialis squatarola* | 2.176 | 1.491 | [52] |
| *Pluvianus aegyptius* | 1.544 | 0.968 | [53] |
| *Ptychoramphus aleuticus* | 2.004 | 1.473 | [54] |
| *Recurvirostra americana* | 2.213 | 1.511 | [19] |
| *Rissa tridactyla* | 2.521 | 1.707 | [23,43,44,55] |
| *Rynchops niger* | 2.155 | 1.430 | [35] |
| *Stercorarius longicaudus* | 2.431 | 1.604 | [56] |
| *Stercorarius maccormicki* | 2.751 | 1.996 | [30,43] |
| *Stercorarius parasiticus* | 2.653 | 1.701 | [5,16] |
| *Sterna forsteri* | 1.903 | 1.328 | [19,57] |
| *Sterna hirundo* | 2.134 | 1.312 | [10,24,38] |
| *Sterna paradisaea* | 2.122 | 1.270 | [5,16,24] |
| *Sternula albifrons* | 1.792 | 0.940 | [24] |
| *Thalasseus maximus* | 2.507 | 1.833 | [40] |
| *Thalasseus sandvicensis* | 2.279 | 1.558 | [40] |
| *Tringa semipalmatus* | 2.387 | 1.604 | [30,58] |
| *Tringa totanus* | 2.072 | 1.322 | [38] |
| *Uria lomvia* | 2.701 | 2.020 | [5,10,16,59,60] |
| *Vanellus miles* | 2.144 | 1.491 | [2] |
| Ciconiiformes | | | |
| *Ardea albus* | 2.364 | 1.687 | [40] |
| *Ardea purpurea* | 2.591 | 1.699 | [30] |
| *Botaurus lentiginosus* | 2.306 | 1.504 | [31] |
| *Bubulcus ibis* | 2.221 | 1.450 | [30,40] |
| *Egretta garzetta* | 2.380 | 1.425 | [30] |
| *Egretta thula* | 2.083 | 1.354 | [10,40] |
| *Egretta tricolor* | 2.100 | 1.425 | [40] |
| *Eudocimus albus* | 2.462 | 1.706 | [40] |
| *Nycticorax nycticorax* | 2.261 | 1.519 | [30,40] |
| *Plegadis falcinellus* | 2.316 | 1.573 | [40] |
| *Plegadis ridgwayi* | 2.481 | 1.530 | [47] |
| Coraciiformes | | | |
| *Merops ornatus* | 1.415 | 0.629 | [61] |
| Falconidae | | | |
| *Falco cenchroides* | 1.699 | 1.276 | [2] |
| *Falco sparverius* | 1.908 | 1.152 | [5,62] |
| *Falco tinnunculus* | 2.230 | 1.326 | [5,63] |
| Galliformes | | | |
| *Alectura lathami* | 2.531 | 2.255 | [64,65] |
| *Centrocercus urophasianus* | 2.322 | 1.580 | [5,66] |
| *Gallus gallus* | 2.687 | 1.752 | [67-69] |
| *Lagopus lagopus* | 2.193 | 1.328 | [70,71] |
| *Lagopus muta* | 2.262 | 1.372 | [72] |
| *Leipoa ocellata* | 2.425 | 2.238 | [64,65] |
| *Phasianus colchicus* | 2.308 | 1.491 | [5,10,43,63,73] |
| Gaviiformes | | | |
| *Gavia stellata* | 2.791 | 1.887 | [74] |
| Gruiformes | | | |
| *Coturnicops noveboracensis* | 1.859 | 0.839 | [75] |
| *Fulica americana* | 2.273 | 1.507 | [76-79] |
| *Gallinula tenebrosa* | 2.274 | 1.524 | [80] |
| *Grus canadensis* | 2.914 | 2.229 | [5,81,82] |
| *Porphyrio porphyrio* | 2.267 | 1.546 | [80] |
| *Porzana carolina* | 1.724 | 0.939 | [31,83] |
| *Rallus elegans* | 2.041 | 1.279 | [84] |
| Passeriformes | | | |
| *Agelaius phoeniceus* | 1.704 | 0.609 | [30,31,85] |
| *Aphelocoma coerulescens* | 1.662 | 0.764 | [86,87] |
| *Calcarius lapponicus* | 1.623 | 0.435 | [88,89] |
| *Calcarius mccownii* | 1.699 | 0.380 | [90] |
| *Carduelis cannabina* | 1.342 | 0.199 | [5,63] |
| *Carduelis tristis* | 1.176 | 0.176 | [30] |
| *Corvus caurinus* | 2.287 | 1.250 | [91] |
| *Delichon urbicum* | 1.301 | 0.281 | [38] |
| *Dendroica petechia* | 1.204 | 0.214 | [5,30,92] |
| *Dumetella carolinensis* | 1.690 | 0.568 | [30] |
| *Emberiza citrinella* | 1.643 | 0.452 | [5,63] |
| *Erithacus rubecula* | 1.531 | 0.382 | [5,63] |
| *Euplectes orix* | 1.220 | 0.238 | [93] |
| *Ficedula hypoleuca* | 1.257 | 0.220 | [94] |
| *Fringilla coelebs* | 1.644 | 0.324 | [5,63] |
| *Gerygone igata* | 1.313 | 0.173 | [95] |
| *Heteromyias albispecularis* | 1.886 | 0.699 | [96] |
| *Hirundo rustica* | 1.293 | 0.302 | [31,85] |
| *Junco hyemalis* | 1.301 | 0.387 | [97] |
| *Lanius ludovicianus* | 1.721 | 0.667 | [98] |
| *Leucosticte australis* | 1.952 | 0.414 | [99] |
| *Melospiza melodia micronyx* | 1.442 | 0.455 | [100] |
| *Menura novaehollandiae* | 2.288 | 1.789 | [101] |
| *Orthonyx spaldingii* | 1.851 | 1.152 | [95] |
| *Passer domesticus* | 1.506 | 0.463 | [30,85,102,103] |
| *Passer moabiticus* | 1.204 | 0.255 | [30] |
| *Passerculus sandwichensis* | 1.524 | 0.338 | [31] |
| *Passerina cyanea* | 1.519 | 0.320 | [104] |
| *Phylloscopus trochilus* | 1.097 | -0.022 | [38] |
| *Plectrophenax nivalis* | 1.681 | 0.501 | [89] |
| *Ploceus capensis* | 1.653 | 0.560 | [105] |
| *Prionodura newtoniana* | 2.090 | 1.061 | [106] |
| *Prunella modularis* | 1.477 | 0.328 | [5,63] |
| *Ptiloris victoriae* | 2.015 | 1.009 | [107] |
| *Quiscalus major* | 1.921 | 0.908 | [108] |
| *Quiscalus quiscula* | 1.787 | 0.827 | [31] |
| *Sayornis nigricans* | 1.383 | 0.322 | [109] |
| *Sayornis phoebe* | 1.301 | 0.312 | [31,85] |
| *Sialia sialis* | 1.486 | 0.464 | [110] |
| *Sturnus vulgaris* | 1.909 | 0.846 | [85] |
| *Sylvia communis* | 1.431 | 0.255 | [5,63] |
| *Tachycineta bicolor* | 1.299 | 0.279 | [85] |
| *Toxostoma rufum* | 1.819 | 0.740 | [111] |
| *Troglodytes aedon* | 1.216 | 0.165 | [112,113] |
| *Turdus iliacus* | 1.826 | 0.777 | [5,114] |
| *Turdus merula* | 1.886 | 0.839 | [5,114] |
| *Turdus migratorius* | 1.843 | 0.805 | [31,85] |
| *Turdus philomelos* | 1.708 | 0.763 | [5,63] |
| *Turdus pilaris* | 2.000 | 0.903 | [38] |
| *Xanthocephalus xanthocephalus* | 1.739 | 0.653 | [115] |
| *Xenicus gilviventris* | 1.557 | 0.407 | [116] |
| Pelecaniformes | | | |
| *Fregata minor* | 2.309 | 1.950 | [49,117] |
| *Morus bassanus* | 2.431 | 2.019 | [118] |
| *Phalacrocorax aristotelis* | 2.366 | 1.683 | [5,16,119] |
| *Sula abbotti* | 2.318 | 2.027 | [120,121] |
| *Sula sula* | 2.249 | 1.766 | [26,49,122] |
| Phaethontidae | | | |
| *Phaethon aethereus* | 2.301 | 1.797 | [121] |
| *Phaethon lepturus* | 2.230 | 1.610 | [123] |
| *Phaethon rubricauda* | 2.299 | 1.855 | [49] |
| Piciformes | | | |
| *Dendrocopos syriacus* | 1.908 | 0.732 | [10] |
| Podicipediformes | | | |
| *Podiceps nigricollis* | 2.245 | 1.340 | [78] |
| *Podilymbus podiceps* | 2.126 | 1.327 | [77,124] |
| Procellariiformes | | | |
| *Bulweria bulwerii* | 1.878 | 1.320 | [49,125,126] |
| *Calonectris diomedea borealis* | 2.483 | 1.986 | [126] |
| *Diomedea exulans* | 3.024 | 2.707 | [30,127,128] |
| *Hydrobates pelagicus* | 1.602 | 0.845 | [30] |
| *Macronectes giganteus* | 2.843 | 2.433 | [127] |
| *Oceanodroma castro* | 1.591 | 0.929 | [126] |
| *Oceanodroma furcata* | 1.563 | 1.097 | [129] |
| *Oceanodroma leucorhoa* | 1.666 | 1.023 | [30,130] |
| *Pachyptila vittata* | 2.068 | 1.544 | [127] |
| *Phoebastria immutabilis* | 2.852 | 2.449 | [131,132] |
| *Phoebastria nigripes* | 2.849 | 2.484 | [131] |
| *Procellaria aequinoctialis* | 2.535 | 2.121 | [127] |
| *Procellaria westlandica* | 2.580 | 2.176 | [133] |
| *Pterodroma alba* | 2.090 | 1.748 | [134] |
| *Pterodroma hypoleuca* | 2.000 | 1.593 | [26,135] |
| *Pterodroma inexpectata* | 2.342 | 1.790 | [26] |
| *Pterodroma phaeopygia* | 2.439 | 1.886 | [136] |
| *Puffinus nativitatis* | 2.110 | 1.695 | [137] |
| *Puffinus pacificus* | 2.178 | 1.764 | [30,138,139] |
| *Puffinus puffinus* | 2.148 | 1.766 | [140] |
| *Puffinus tenuirostris* | 2.431 | 1.942 | [141] |
| Sphenisciformes | | | |
| *Aptenodytes patagonicus* | 2.867 | 2.481 | [10,142] |
| *Spheniscus demersus* | 2.614 | 2.003 | [10] |
| *Pygoscelis adeliae* | 2.630 | 2.096 | [43,143] |
| Strigiformes | | | |
| *Aegolius funereus* | 1.908 | 1.101 | [5,144] |
| *Asio otus* | 2.209 | 1.405 | [1] |
| *Bubo virginianus* | 2.284 | 1.729 | [1] |
| *Megascops asio* | 2.087 | 1.236 | [145,146] |
| *Tyto alba* | 2.079 | 1.326 | [1,5,10] |
| Struthioniformes | | | |
| *Apteryx australis* | 2.815 | 2.615 | [147] |
| *Dromaius novaehollandiae* | 3.031 | 2.828 | [148] |
| *Struthio camelus* | 3.627 | 3.139 | [149-151] |
| Crocodylia | | | |
| *Crocodylus acutus* | 2.135 | 1.963 | [152,153] |

**References**

1. Sumner FL (1929) Comparative studies in the growth of young raptors. Condor 31: 85-111.

2. Marchant S, Higgins PJ (1994) Handbook of Australian, New Zealand and Antarctic Birds. Volume 2: Raptors to Lapwings. Oxford, United Kingdom: Oxford University Press. 1048 p.

3. Sumner EL (1929) Notes on the growth and behavior of young Golden Eagles. Auk 46: 161-169.

4. Groebbles F (1927) Oologische Studien. I. Mitt. Untersuchungen über Gewichte, spezifische Gewichte und Zusammensetzung von Vogeleiern verschiedener Bebrütungsstadien. Journal of Ornithology 75: 225-235.

5. Drent RH (1970) Functional aspects of incubation in the Herring Gull. Behaviour Supplement 17: 1-132.

6. Sykes PW (1987) Some aspects of the breeding biology of the Snail Kite in Florida. Journal of Field Ornithology 58: 171-189.

7. Carey C, Leon-Velarde F, Dunin-Borkowskit O, Monge C (1989) Shell conductance, daily water loss, and water content of Puna Teal eggs. Physiological Zoology 62: 83-95.

8. Ely CR, Raveling DG (1984) Breeding biology of Pacific White-fronted Geese. Journal of Wildlife Management 48: 823-837.

9. Ely CR, Dzubin AX (1994) Greater White-fronted Goose (*Anser albifrons*). In: Poole A, Gill F, editors. The Birds of North America, No 131: The Academy of Natural Sciences, Philadelphia, and the American Ornithologists' Union, Washington, D.C. pp. 1-32.

10. Ar A, Rahn H (1985) Pores in avian eggshells: gas conductance, gas exchange and embryonic growth rate. Respiration Physiology 61: 1-20.

11. Eadie JM, Mallory ML, Lumsden HG (1995) Common Goldeneye (*Bucephala clangula*). In: Poole A, Gill F, editors. The Birds of North America, No 170: The Academy of Natural Sciences, Philadelphia, and the American Ornithologists' Union, Washington, D.C. pp. 1-32.

12. Eadie JM, Savard J-PL, Mallory ML (2000) Barrow's Goldeneye (*Bucephala islandica*). In: Poole A, Gill F, editors. The Birds of North America, No 548. Philadelphia: The Birds of North America. pp. 1-32.

13. Booth DT, Sotherland PR (1991) Oxygen consumption, air-cell gas tensions and incubation parameters of Mute Swan eggs. Physiological Zoology 64: 473-484.

14. Dugger BD, Dugger KM, Fredrickson LH (1994) Hooded Merganser (*Lophodytes cucullatus*). In: Poole A, Gill F, editors. The Birds of North America, No 98: The Academy of Natural Sciences, Philadelphia, and the American Ornithologists' Union, Washington, D.C. pp. 1-24.

15. Gross AO (1938) Eider ducks of Kent's Islands. Auk 55: 387-4000.

16. Belopol'skii LO (1961) Ecology of sea colony birds of the Barents Sea. Jerusalem: Israel Program for Scientific Translations. 346 p.

17. Hagelund K, Norderhaug M (1973) Studies of population changes and breeding processes in a colony of Eiders (*Somateria mollissima* (L.)) in Svalbard. Norsk Polarinst Årbok 1973: 141-161.

18. Lowther PE, Collins CT (2002) Black Swift (*Cypseloides niger*). In: Poole A, Gill F, editors. The Birds of North America, No 676. Philadelphia: The Birds of North America. pp. 1-16.

19. Grant GS (1982) Avian incubation: egg temperature, nest humidity, and behavioral thermoregulation in a hot environment. Ornithological Monographs 30: 1-75.

20. Lank DB, Oring LW, Maxson SJ (1985) Mate and nutrient limitation of egg-laying in a polyandrous shorebird. Ecology 66: 1513-1524.

21. Plumb WJ (1965) Observations on the breeding biology of the Razorbill. British Birds 58: 449-456.

22. Stempniewicz L (1981) Breeding biology of the Little Auk *Plautus alle* in the Hornsund region, Spitsbergen. Acta Ornithologica 18: 1-26.

23. Rahn H, Paganelli CV (1990) Gas fluxes in avian eggs: driving forces and the pathway for exchange. Comparative Biochemistry and Physiology A 95: 1-15.

24. Rahn H, Paganelli CV, Nisbet ICT, Whittow GC (1976) Regulation of incubation water loss in eggs of seven species of terns. Physiological Zoology 49: 245-259.

25. Morris RD, Chardine JW (1992) The Breeding biology and aspects of the feeding ecology of Brown Noddies *Anous stolidus* nesting near Culebra, Puerto-Rico, 1985-1989. Journal of Zoology 226: 65-79.

26. Whittow GC (1980) Physiological and ecological correlates of prolonged incubation in sea birds. American Zoologist 20: 427-436.

27. Andreev AV, Golubova EY (1995) A new finding of breeding Kittlitz's murrelet *Brachyramphus brevirostris* on the Ochotsk Sea coast. Russian Journal of Ornithology 4: 63-64.

28. Klima J, Jehl JJR (1998) Stilt Sandpiper (*Calidris himantopus*). In: Poole A, Gill F, editors. The Birds of North America, No 341. Philadelphia: The Birds of North America. pp. 1-20.

29. Asbirk S (1979) The adaptive significance of the reproductive pattern in the Black Guillemot, *Cepphus grylle*. Videnskaplige Meddelelser Dansk naturhistorisk Forening 141: 29-80.

30. Ar A, Rahn H (1980) Water in the avian egg: overall budget of incubation. American Zoologist 20: 373-384.

31. Manning TH (1981) Analysis of weight lost by Eegs of 11 species of birds during Incubation. Canadian Field-Naturalist 95: 63-68.

32. Davis TA, Ackerman RA (1985) Adaptations of Black Tern (*Chilidonias niger*) eggs for water loss in a moist nest. Auk 102: 640-643.

33. Piatt JF, Kitaysky AS (2002) Tufted Puffin (*Fratercula cirrhata*). In: Poole A, Gill F, editors. The Birds of North America, No 708. Philadelphia: The Birds of North America. pp. 1-32.

34. Piatt JF, Kitaysky AS (2002) Horned Puffin (*Fratercula corniculata*). In: Poole A, Gill F, editors. The Birds of North America, No 603. Philadelphia: The Birds of North America. pp. 1-28.

35. Grant GS, Paganelli CV, Rahn H (1984) Microclimate of Gull-billed Tern and Black Skimmer nests. Condor 86: 337-338.

36. Pettit TN, Grant GS, Whittow GC, Rahn H, Paganelli CV (1981) Respiratory gas exchange and growth of white tern embryos. Condor 83: 355-361.

37. Andres BA, Falxa GA (1995) Black Oystercatcher (*Haematopus bachmani*). In: Poole A, Gill F, editors. The Birds of North America, No 155: The Academy of Natural Sciences, Philadelphia, and the American Ornithologists' Union, Washington, D.C. pp. 1-20.

38. Barth EK (1953) Calculation of egg volume based on loss of weight during Incubation. Auk 70: 151-159.

39. Harris MP (1964) Aspects of the breeding biology of the gulls. Ibis 106: 432-456.

40. Vleck CM, Vleck D, Rahn H, Paganelli CV (1983) Nest microclimate, water-vapor conductance, and water-loss in heron and tern eggs. Auk 100: 76-83.

41. Fordham RA (1964) Breeding biology of the southern Black-backed Gull I: pre-egg and egg stage. Notornis 11: 3-34.

42. Fordham RA (1964) Breeding biology of the southern Black-backed Gull II: incubation and the chick stage. Notornis 11: 110-126.

43. Rahn H, Ackerman RA, Paganelli CV (1977) Humidity in avian nest and egg water loss during incubation. Physiological Zoology 50: 269-283.

44. Morgan KR, Paganelli CV, Rahn H (1978) Egg weight loss and nest humidity during incubation in two Alaskan gulls. Condor 80: 272-275.

45. Rahn H, Dawson WR (1979) Incubation water loss in eggs of Heermann's and Western Gulls. Physiological Zoology 52: 451-460.

46. Wooller RD, Dunlop JN (1980) The use of simple measurements to determine the age of Silver Gull eggs. Australian Wildlife Research 7: 113-115.

47. Carey C, Leonvelarde F, Castro G, Monge C (1987) Shell conductance, daily water loss, and water content of Andean Gull and Puna Ibis eggs. Journal of Experimental Zoology: 247-252.

48. Skeel MA, Mallory EP (1996) Whimbrel (*Numenius phaeopus*). In: Poole A, Gill F, editors. The Birds of North America, No 219: The Academy of Natural Sciences, Philadelphia, and the American Ornithologists' Union, Washington, D.C. pp. 1-28.

49. Whittow GC (1983) Physiological ecology of incubation in tropical seabirds. Studies in Avian Biology 8: 47-72.

50. Whittow GC, Grant GS, Flint EN (1985) Egg water loss, shell water-vapor conductance, and the incubation period of the Gray-backed Tern (*Sterna lunata*). Condor 87: 269-272.

51. Tracy DM, Schamel D, Dale J (2002) Red Phalarope (*Phalaropus fulicarius*). In: Poole A, Gill F, editors. The Birds of North America, No 698. Philadelphia: The Birds of North America. pp. 1-32.

52. Hussell DJT, Page GW (1976) Observations on breeding biology of Black-bellied Plovers on Devon-Island, NWT, Canada. Wilson Bulletin 88: 632-653.

53. Howell TR (1979) Breeding Biology of the Egyptian Plover, *Pluvianus aegyptinus*. Berkeley: University of California Press. 76 p.

54. Roudybush T, Hoffman L, Rahn H (1980) Conductance, pore geometry, and water loss of eggs of Cassin's Auklet. Condor 82: 105-106.

55. Maunder JE, Threlfall W (1972) The breeding biology of the Black-legged Kittiwake in Newfoundland. Auk 89: 789-816.

56. Wiley RH, Lee DS (1998) Long-tailed Jaeger (*Stercorarius longicaudus*). In: Poole A, Gill F, editors. The Birds of North America, No 365. Philadelphia: The Birds of North America. pp. 1-24.

57. McNicholl MK, Lowther PE, Hall JA (2001) Forster's Tern (*Sterna forsteri*). In: Poole A, Gill F, editors. The Birds of North America, No 595. Philadelphia: The Birds of North America. pp. 1-24.

58. Raynor GS, Wilcox L (1980) Observations on the life-history of Willets on Long-Island, New-York. Wilson Bulletin 92: 253-258.

59. Gaston AJ, Nettleship DN (1981) The thick-billed Murres of Prince Leopold Island: a study of the breeding ecology of a colonial high arctic seabirds. Ottawa, Canada: Canadian Wildlife Service. 350 p.

60. Uspenski SM (1958) The bird Bazaars of Novaya Zemlya. Montreal, Canada: Canadian Wildlife Service.

61. Lill A, Fell PJ (2007) Microclimate of nesting burrows of the Rainbow Bee-eater. Emu 107: 108-114.

62. Sherman AR (1913) The nest life of the Sparrow Hawk. Auk 30: 406-418.

63. Groebbles F, Mobert F (1927) Oologische studien. II. Mitteilupg. Ueber die kunstiiche bestimmung der brutdauer einiger vogelarten mit hesonderser berucksichtigung des eigewichts. Journal of Ornithology 17: 198-205.

64. Seymour RS, Vleck D, Vleck CM, Booth DT (1987) Water relations of buried eggs of mound building birds. Journal of Comparative Physiology B 157: 413-422.

65. Vleck D, Vleck CM, Seymour RS (1984) Energetics of embryonic development in the megapode birds, Mallee Fowl *Leipoa ocellata* and Brush Turkey *Alectura lathami*. Physiological Zoology 57: 444-456.

66. Patterson RL (1952) The Sage Grouse in Wyoming. Denver: Sage Books Wyoming Game and Fish Commission. 341 p.

67. Burke E (1925) A study of incubation. Bulletin of University of Montana Agricultural Experimental Station 178: 1-43.

68. Chattock AP (1925) On the physics of incubation. Philosophical Transactions of the Royal Society of London Series B 213: 397-450.

69. Horton DH (1932) The loss of weight of duck eggs during incubation. Poultry Science 11: 23-27.

70. Andersen O, Steen JB (1986) Water economy in bird nests. Journal of Comparative Physiology B 156: 823-828.

71. Steen JB, Andersen O, Saebo A, Pedersen HC, Erikstad KE (1988) Viability of newly hatched chicks of Willow Ptarmigan *Lagopus l. lagopus*. Ornis Scandinavica 19: 93-96.

72. Steen JB, Unander S (1985) Breeding biology of the Svalbard Rock Ptarmigan *Lagopus mutus hyperboreus*. Ornis Scandinavica 16: 191-197.

73. Gladstone HS (1904) Note on the decrease in the weight of eggs as incubation advances. Ibis 28: 376.

74. Barr JF, Eberl C, McIntyre JW (2000) Red-throated Loon (*Gavia stellata*). In: Poole A, Gill F, editors. The Birds of North America, No 513. Philadelphia: The Birds of North America. pp. 1-28.

75. Elliot RD, Morrison RIG (1979) Incubation period of the Yellow Rail. Auk 96: 422-423.

76. Gullion GW (1954) The reproductive cycle of American Coots in California. Auk 71: 366-412.

77. Davis TA, Platter-Reiger MF, Ackerman RA (1984) Incubation water loss by Pied-billed Grebe: eggs adaptation to a hot wet nest. Physiological Zoology 57: 384-391.

78. Sotherland PR, Ashen MD, Shuman RD, Tracy CR (1984) The water balance of bird eggs incubated in water. Physiological Zoology 57: 338-348.

79. Carey C, Leon-Velarde F, Dunin-Borkowski O, Bucher TL, de La Torre G, et al. (1989) Variation in eggshell characteristics and gas exchange of montane and lowland coot eggs. Journal of Comparative Physiology B 159: 389-400.

80. Lill A (1990) Water vapor flux in the eggs of two species of rail (Rallidae) during incubation. Proceedings of the Royal Society of Victoria 102: 67-70.

81. Walkinshaw LH (1950) The Sandhill Crane in the Bernard W. Baker Sanctuary, Michigan. Auk 67: 38-51.

82. Walkinshaw LH (1950) Incubation period of the Sandhill Crane, *Grus canadensis tabida*. Auk 67: 513.

83. Walkinshaw LH (1940) Summer Life of the Sora Rail. Auk 57: 153-168.

84. Meanley B (1969) Natural history of the King Rail. Washington D.C.: United States Department of the Interior Bureau of Sport fisheries and Wildlife. 108 p.

85. Manning TH (1982) Daily measurements of variation in weight-loss of eggs of seven Passerine species before and during natural incubation. Canadian Journal of Zoology 60: 3143-3149.

86. Woolfenden GE, Fitzpatrick JW (1996) Florida Scrub-Jay (*Aphelocoma coerulescens*). In: Poole A, Gill F, editors. The Birds of North America, No 228: The Academy of Natural Sciences, Philadelphia, and the American Ornithologists' Union, Washington, D.C. pp. 1-28.

87. Woolfenden GE (1978) Growth and survival of Young Florida Scrub Jays. Wilson Bulletin 90: 1-18.

88. Hussell DJT, Montgomerie R (2002) Lapland Longspur (*Calcarius lapponicus*). In: Poole A, Gill F, editors. The Birds of North America, No 656. Philadelphia: The Birds of North America. pp. 1-32.

89. Hussell DJT (1972) Factors affecting clutch size in Arctic Passerines. Ecological Monographs 42: 317-364.

90. Mickey FW (1943) Breeding habits of Mccown's Longspur. Auk 60: 181-209.

91. Butler RW, Verbeek NAM, Richardson H (1984) The breeding biology of the Northwestern Crow. Wilson Bulletin 96: 408-418.

92. Schrantz FG (1943) Nest life of the Eastern Yellow Warbler. Auk 60: 367-387.

93. Woodall PF, Parry DF (1982) Water-loss during incubation in Red Bishop (*Euplectes orix*) eggs. South African Journal of Zoology 17: 75-78.

94. Kern MD, Cowie RJ, Yeager M (1992) Water loss, conductance, and structure of eggs of Pied Flycatchers during egg laying and incubation. Physiological Zoology 65: 1162-1187.

95. Higgins PJ, Peter JM (2002) Handbook of Australian, New Zealand and Antarctic birds. Volume 6: pardalotes to shrike-thrushes. Oxford, United Kingdom: Oxford University Press. 1262 p.

96. Frith DW, Frith CB (2000) The nesting biology of the Grey-headed Robin *Heteromyias albispecularis* (Petroicidae) in Australian upland tropical rainforest. Emu 100: 81-94.

97. Nolan VJ, Ketterson ED, Cristol DA, Rogers CM, Clotfelter ED, Titus RC, et al. (2002) Dark-eyed Junco (*Junco hyemalis*). In: Poole A, Gill F, editors. The birds of North America, 716. Philadelphia: The Birds of North America.

98. Miller AH (1931) Systematic revision and natural history of the American Shrikes (*Lanius*). University of California Publications in Zoology 38: 11-242.

99. Johnson RE, Hendricks P, Pattie DL, Hunter KB (2000) Brown-capped Rosy-Finch (*Leucosticte australis*). In: Poole A, Gill F, editors. The Birds of North America, No 536. Philadelphia: The Birds of North America. pp. 1-24.

100. Kern MD, Sogge MK, Vanriper C (1990) Water-vapor pressure in nests of the San-Miguel Island Song Sparrow. Condor 92: 761-767.

101. Lill A (1987) Water vapor conductance and shell characteristics of Superb Lyrebird eggs. Australian Journal of Zoology 35: 553-558.

102. Weaver RL (1943) Reproduction in English Sparrows. Auk 60: 62-74.

103. Dawson DG (1964) The eggs of the House Sparrow. Notornis 11: 187-189.

104. Morgan FD (1976) Nesting studies of the Indigo Bunting (*Passerina cyanea*) at Thornhill, Indiana. Proceedings of the Indiana Academy of Science 86: 461-465.

105. Brown CR (1994) Nest microclimate, egg temperature, egg water loss, and eggshell conductance in Cape Weavers *Ploceus capensis*. Ostrich 65: 26-31.

106. Frith CB, Frith DW (1998) Nesting biology of the Golden Bowerbird *Prionodura newtoniana* endemic to Australian upland tropical rainforest. Emu 98: 245-268.

107. Frith CB, Frith DW (1995) Notes on the nesting biology and diet of Victoria's Riflebird *Ptiloris victoriae*. Emu 95: 162-174.

108. Post WJ, Poston JP, Bancroft GT (1996) Boat-tailed Grackle (*Quiscalus major*). In: Poole A, Gill F, editors. The Birds of North America, No 207: The Academy of Natural Sciences, Philadelphia, and the American Ornithologists' Union, Washington, D.C. pp. 1-28.

109. Wolf BO (1997) Black Phoebe (*Sayornis nigricans*). In: Poole A, Gill F, editors. The Birds of North America, No 268: The Academy of Natural Sciences, Philadelphia, and the American Ornithologists' Union, Washington, D.C. pp. 1-20.

110. Hamilton WJ (1943) Nesting of the Eastern Bluebird. Auk 60: 91-94.

111. Cavitt JF, Haas CA (2000) Brown Thrasher (*Toxostoma rufum*). In: Poole A, Gill F, editors. The Birds of North America, No 557. Philadelphia: The Birds of North America. pp. 1-28.

112. Kendeigh SC (1940) Factors affecting length of incubation. Auk 57: 499-513.

113. Kendeigh SC (1963) Thermodynamics of incubation in the House Wren, *Troglodytes aedon*. Proceedings of the International Ornithological Congress 13: 884-904.

114. Foster NH (1902) Observations on the weights of birds' eggs. The Irish Naturalist 1902: 237-245.

115. Twedt DJ, Crawford RD (1995) Yellow-headed Blackbird (*Xanthocephalus xanthocephalus*). In: Poole A, Gill F, editors. The birds of North America, No 192: The Academy of Natural Sciences, Philadelphia, and The American Ornithologists' Unnion, Washington, DC.

116. Higgins PJ, Peter JM, Steele WK (2001) Handbook of Australian, New Zealand and Antarctic Birds, vol. 5. Tyrant-flycatchers to chats. Melbourne, Australia: Oxford University Press.

117. Whittow GC, Grant GS, Pettit TN (2003) Water loss from eggs of the Great Frigatebird. Wilson Bulletin 115: 99-101.

118. Nelson JB (1966) Breeding biology of Gannet *Sula bassana* on Bass Rock Scotland. Ibis 108: 584-&.

119. Snow B (1960) The breeding biology of the Shag *Phalacrocorax aristotelis* on the Island of Lundy, Bristol Channel. Ibis 102: 554-575.

120. Nelson JB (1971) Biology of Abbotts Booby *Sula abbotti*. Ibis 113: 429-&.

121. Nelson JB (2006) Pelicans, cormorants and their relatives. Oxford, United Kingdom: Oxford University Press.

122. Whittow GC, Pettit TN, Ackerman RA, Paganelli CV (1989) The regulation of water-loss from the eggs of the Red-footed Booby (*Sula sula*). Comparative Biochemistry and Physiology A 93: 807-810.

123. Stonehouse B (1962) The Tropic Birds (Genus *Phaethon*) of Ascension Island. Ibis 103: 124-161.

124. Ackerman RA, Platter-Rieger M (1979) Water loss by Pied-billed Grebe (*Podilymbus podiceps*) eggs. American Zoologist 19: 921.

125. Whittow GC, Pettit TN (2000) Egg dimensions and shell characteristics of Bulwer's Petrels, *Bulweria bulwerii*, on Laysan Island, northwestern Hawaiian islands. Pacific Science 54: 183-188.

126. Robertson HA, James PC (1988) Morphology and egg measurements of seabirds breeding on Great Salvage Island, North Atlantic. British Ornithologists' Club 108: 79-87.

127. Brown CR, Adams NJ (1988) Egg temperature, embryonic metabolism, and water loss from the eggs of subantarctic Procellariiformes. Physiological Zoology 61: 126-136.

128. Tickell WLN (1968) The biology of the Great Albatrosses, *Diomedea exulans* and *Diomedea epomophora*. Antarctic Research Series 12: 1-55.

129. Boersma PD, Wheelwright NT (1979) Egg neglect in the Procellariiformes: reproductive adaptations in the Fork-tailed Storm Petrel. Condor 81: 157-165.

130. Rahn H, Huntington CE (1988) Eggs of Leach's Storm Petrel: O_2_ uptake, water loss, and microclimate of the nest. Comparative Biochemistry and Physiology A 91: 519-521.

131. Grant GS, Pettit TN, Rahn H, Whittow GC, Paganelli CV (1982) Water loss from Laysan and Black-footed Albatross eggs. Physiological Zoology 55: 405-414.

132. Fisher HI (1969) Eggs and egg-laying in the Laysan Albatross, *Diomedea immutabilis*. Condor 71: 102-112.

133. Baker AJ, Coleman JD (1977) The breeding cycle of the Westland Black Petrel (*Procellaria westlandica*). Notornis 24: 211-231.

134. Rahn H, Whittow GC (1988) Adaptations to a pelagic life: eggs of the Albatross, Shearwater and Petrel. Comparative Biochemistry and Physiology A 91: 415-423.

135. Grant GS, Pettit TN, Rahn H, Whittow GC, Paganelli CV (1982) Regulation of water-loss from Bonin Petrel (*Pterodroma hypoleuca*) eggs. Auk 99: 236-242.

136. Whittow GC, Simons TR, Pettit TN (1984) Water loss from the eggs of a tropical sea bird (*Pterodroma phaeopygia*) at high altitude. Comparative Biochemistry and Physiology A 78: 537-540.

137. Whittow GC (2001) Incubation weight loss of Christmas Shearwater eggs on Christmass Island, Pacific Ocean. Notornis 48: 175-176.

138. Whittow GC, Ackerman RA, Paganelli CV, Pettit TN (1982) Pre-pipping water loss from the eggs of the Wedge-tailed Shearwater. Comparative Biochemistry and Physiology A 72: 29-34.

139. Ackerman RA, Whittow GC, Paganelli CV, Pettit TN (1980) Oxygen consumption, gas exchange, and growth of embryonic Wedge-tailed Shearwaters (*Puffinus pacificus chlororhynchus*). Physiological Zoology 53: 210-221.

140. Harris MP (1966) Breeding biology of the Manx Shearwater *Puffinus puffinis*. Ibis 108: 17-33.

141. Fitzherbert K (1985) The role of energetic factors in the evolution of the breeding biology of the Short-tailled Shearwater (*Puffinus tenuirostris*, Temminck) [Ph.D.]. Victoria: Monash University. 225 p.

142. Handrich Y (1989) Incubation water loss in King Penguin egg .I. Change in egg and brood pouch parameters. Physiological Zoology 62: 96-118.

143. Rahn H, Hammel HT (1982) Incubation water loss, shell conductance, and pore dimensions in Adelie Penguin eggs. Polar Biology 1: 91-97.

144. Kuhk R (1949) Aus der Fortpflanzungsbiologie des Rauhfusskauzes, *Aegolius funereus* (L.). In: Mayr E, Schuz E, editors. Ornithologie als biologische Wissenschaft. Heidelberg: Carl Winter Universitatsverlag. pp. 171-182.

145. Sherman AR (1911) Nest life of the Screech Owl. Auk 28: 155-168.

146. Sumner EL (1928) Notes on the development of young Screech Owls. Condor 30: 333-338.

147. Colbourne R (2002) Incubation behaviour and egg physiology of Kiwi (*Apteryx* spp.) in natural habitats. New Zealand Journal of Ecology 26: 129-138.

148. Buttemer WA, Astheimer LB, Dawson TJ (1988) Thermal and water relations of emu eggs during natural incubation. Physiological Zoology 61: 483-494.

149. Bertram BCR, Burger AE (1981) Aspects of incubation in ostrich. Ostrich 52: 36-43.

150. Swart D, Rahn H, Dekock J (1987) Nest microclimate and incubation water loss of eggs of the African ostrich (*Struthio camelus* var. *domesticus*). Journal of Experimental Zoology: 239-246.

151. Swart D, Rahn H (1988) Microclimate of ostrich nests: measurements of egg temperature and nest humidity using egg hygrometers. Journal of Comparative Physiology B 157: 845-854.

152. Moore JC (1953) The crocodile in the Everglades National-Park. Copeia 1953: 54-59.

153. Lutz PL, Dunbar-Cooper A (1984) The nest environment of the American Crocodile (*Crocodylus acutus*). Copeia 1984: 153-161.
