# Supplementary material for: Eggshell Porosity Provides Insight on Evolution of Nesting in Dinosaurs
Source: PLoS One. 2015 Nov 25;10(11):e0142829. doi: 10.1371/journal.pone.0142829 (PMC4659668; doi:10.1371/journal.pone.0142829)
Supplement: S2 Table — (DOCX) [file pone.0142829.s007.docx]

**S2 Table. Museum specimens assessed for eggshell porosity in this study**.

| Specimen | Taxon | Collection number |
| --- | --- | --- |
| Modern eggshell | *Aix galericulata* | UHR 33131 |
|  | *Aix sponsa* | UHR 33128 |
|  | *Alectura lathami* | ZEC 137-1-3; ZEC 137-1-4; ZEC 137-1-5 |
|  | *Alligator mississippiensis* | YPM HERR. 015109; YPM HERR. 015110 |
|  | *Alligator sinensis* | YPM HERR. 018989 |
|  | *Ammoperdix heyi* | ROM 9516 |
|  | *Anas bahamensis* | ROM 9487 |
|  | *Anas discors* | ROM 7563 |
|  | *Anas platyrhynchos* | ROM 12821 |
|  | *Anhinga anhinga* | ROM 9438 |
|  | *Anser anser* | ZEC 221-1-2; ZEC 221-1-3 |
|  | *Branta canadensis* | ZEC 444-1-1; ZEC 444-1-2 |
|  | *Bucephala islandica* | ROM 7739 |
|  | *Burhinus oedicnemus* | ROM 10983 |
|  | *Buteo rufinus* | ROM 8126 |
|  | *Caiman crocodilus* | UHR 33214 |
|  | *Caiman latirostris* | UHR 27367; YPM HERR. 017953; YPM HERR. 018990 |
|  | *Caiman yacare* | UHR 33215; YPM HERR. 019030 |
|  | *Cairina moschata* | ZEC 290-1-1; ZEC 290-1-2 |
|  | *Chrysolophus amherstiae* | ROM 3659 |
|  | *Clangula hyemalis* | ROM 12427 |
|  | *Crocodylus moreletii* | YPM HERR. 018979; YPM HERR. 018980; YPM HERR. 018981 |
|  | *Crocodilus niloticus* | MCZ 26933; YPM HERR. 017955; YPM HERR. 018982; YPM HERR. 018983; YPM HERR. 018984; YPM HERR. 018985; YPM HERR. 018986; ZEC 136 (HEC175) |
|  | *Crocodylus porosus* | UHR 33210; YPM R17954; YPM R17976 |
|  | *Crocodylus rhombifer* | YPM HERR. 011637; YPM HERR. 015102 |
|  | *Crocodilus siamensis* | YPM HERR. 018977; YPM HERR. 018978 |
|  | *Egretta thula* | ROM 4772 |
|  | *Egretta tricolor* | ROM 4782 |
|  | *Eudocimus albus* | ROM 4818 |
|  | *Eudromia elegans* | ZEC 283-1-2 |
|  | *Falco naumanni* | ROM 5096 |
|  | *Falco tinnunculus* | ROM 10855 |
|  | *Fratercula arctica* | ROM 2354 |
|  | *Fulmarus glacialis* | ROM 3034 |
|  | *Gavialis gangeticus* | YPM HERR. 018824 |
|  | *Larus glaucescens* | ROM 5503 |
|  | *Larus heermanni* | ROM 8686 |
|  | *Larus ridibundus* | ROM 3780 |
|  | *Leipoa ocellata* | ZEC 218-2-1; ZEC218-2-2 |
|  | *Lophura nycthemera* | ROM 4622 |
|  | *Megapodius decollatus* | YPM 142019 |
|  | *Melanosushus niger* | CM41452 (ZEC303); MCZ 46554 |
|  | *Nycticorax nycticorax* | UHR 33121 |
|  | *Oceanodroma leucorhoa* | ROM 2796 |
|  | *Onychoprion fuscatus* | ROM 5632 |
|  | *Osteolaemus tetraspis* | YPM HERR. 018823; YPM HERR. 018988 |
|  | *Paleosuchus palpebrosus* | CM41453 (ZEC304); YPM HERR. 017952 |
|  | *Paleosuchus trigonatus* | YPM HERR. 018987 |
|  | *Passer domesticus* | ROM 2377; ZEC 455-1-1; ZEC 455-1-2 |
|  | *Pavo cristatus* | UHR 33126 |
|  | *Phalacrocorax pelagicus* | ZEC 445-1-1; ZEC 445-1-2 |
|  | *Plegadis falcinellus* | ROM 8002 |
|  | *Pygoscelis adeliae* | ROM 11421 |
|  | *Rissa tridactyla* | ROM 356 |
|  | *Rynchops niger* | ROM 10003 |
|  | *Somateria m. mollissima* | ROM 10863 |
|  | *Spheniscus demersus* | ROM 9973 |
|  | *Sterna paradisaea* | ROM 5615 |
|  | *Sternula albifrons* | ROM 10864 |
|  | *Streptopelia turtur* | ROM 3665 |
|  | *Strix aluco* | ROM 3767 |
|  | *Syrmaticus soemmerringii* | UHR 33127 |
|  | *Tadorna tadorna* | ROM 9527 |
|  | *Thalasseus elegans* | ROM 13086 |
|  | *Thalasseus maximus* | ROM 5660 |
|  | *Tomistoma schlegelii* | YPM HERR. 018975; YPM HERR. 018976 |
|  | *Tyto alba* | ROM 12631 |
| Fossil/sub-fossil eggshell | *Euryapteryx* sp. | AIM LB6672; AIM LB6673 [1,2] |
|  | *Pachyornis geranoides* | AIM LB6675 [1,2] |
|  | *Prismatoolithus levis*/ *Troodon formosus* | TMP1994.179.1 (Holotype: [3]) |

Institutional abbreviations: AIM, Auckland Institute and Museum, Auckland, New Zealand; CM, Carnegie Museum of Natural History, Pittsburgh, Pennsylvania; HEC, Hirsch Egg Catalogue, University of Colorado Museum, Boulder, Colorado; MCZ, Museum of Comparative Zoology, Cambridge, Massachusetts; ROM, Royal Ontario Museum, Toronto, Canada; TMP, Royal Tyrrell Museum of Palaeontology, Drumheller, Canada; UHR, Hokkaido University Museum, Sapporo, Japan; YPM R., Herpetology Collection at the Yale Peabody Museum, New Haven, Connecticut; ZEC, Zelenitsky Egg catalogue, University of Calgary, Calgary, Canada.

**References**

1. Gill BJ (2000) Morphometrics of moa eggshell fragments (Aves: Dinornithiformes) from Late Holocene dune-sands of the Karikari Peninsula, New Zealand. Journal of the Royal Society of New Zealand 30: 131-145.

2. Huynen L, Gill BJ, Millar CD, Lambert DM (2010) Ancient DNA reveals extreme egg morphology and nesting behavior in New Zealand's extinct moa. Proceedings of the National Academy of Sciences of the United States of America 107: 16201-16206.

3. Zelenitsky DK, Hills LV (1996) An egg clutch of *Prismatoolithus levis* oosp. nov. from the Oldman Formation (Upper Cretaceous), Devil's Coulee, southern Alberta. Canadian Journal of Earth Sciences 33: 1127-1131.
