# Supplementary material for: Eggshell Porosity Provides Insight on Evolution of Nesting in Dinosaurs
Source: PLoS One. 2015 Nov 25;10(11):e0142829. doi: 10.1371/journal.pone.0142829 (PMC4659668; doi:10.1371/journal.pone.0142829)
Supplement: S3 Table — (DOCX) [file pone.0142829.s008.docx]

**S3 Table.** Eggshell porosity (**A_p_∙L_s_^-1^**) and egg mass (M) of living and extinct archosaurs. *Eggshell thickness was averaged between unincubated and hatched eggshells [1,2]. †Single pore area was calculated from pore radius reported by Lill [3], assuming that cross-sectional pore canal is round. Egg length and breadth were taken from Schonwetter [4].

| Taxon | log A_p_∙L_s_^-1^ | | | | log M | References |
| --- | --- | --- | --- | --- | --- | --- |
| Accipitridae | | | | | | |
| *Buteo rufinus* | | 0.012 | | | 1.783 | [5]; this study |
| Anseriformes | | | | | | |
| *Aix galericulata* | | 0.590 | | | 1.547 | [4,6,7]; this study |
| *Aix sponsa* | | 0.711 | | | 1.642 | [4,6,7]; this study |
| *Anas bahamensis* | | 0.310 | | | 1.543 | [4,7]; this study |
| *Anas discors* | | -0.259 | | | 1.405 | [4,7]; this study |
| *Anas fulvigula* | | 1.051 | | | 1.747 | [7] |
| *Anas gracilis* | | 0.527 | | | 1.515 | [7] |
| *Anas platyrhynchos* | | 0.537 | | | 1.895 | [4,8,9]; this study |
| *Anser anser* | | 0.857 | | | 2.192 | [4,6-8]; this study |
| *Anser brachyrhynchus* | | 0.817 | | | 2.144 | [7] |
| *Anser cygnoides* | | 1.264 | | | 2.166 | [7] |
| *Anser erythropus* | | 0.902 | | | 2.090 | [7] |
| *Anser fabalis* | | 0.813 | | | 2.183 | [4,6,7] |
| *Branta canadensis* | | 1.138 | | | 2.167 | [4-6]; this study |
| *Branta h. minima* | | 0.383 | | | 2.002 | [7] |
| *Branta leucopsis* | | 0.651 | | | 2.028 | [4,6,7] |
| *Branta sandvicensis* | | 0.905 | | | 2.189 | [4,6,7] |
| *Bucephala islandica* | | 0.822 | | | 1.827 | [7]; this study |
| *Cairina moschata* | | 0.742 | | | 1.898 | [4-6]; this study |
| *Cereopsis novaehollandiae* | | 0.368 | | | 2.127 | [4-6] |
| *Chloephaga melanoptera* | | 0.728 | | | 2.049 | [10] |
| *Clangula hyemalis* | | 0.284 | | | 1.661 | [11]; this study |
| *Cyanochen cyanoptera* | | 0.663 | | | 1.918 | [7] |
| *Dendrocygna arborea* | | 0.703 | | | 1.777 | [7] |
| *Dendrocygna autumnalis* | | 0.352 | | | 1.631 | [7] |
| *Dendrocygna bicolor* | | 0.587 | | | 1.736 | [7] |
| *Lophodytes cucullatus* | | 0.265 | | | 1.718 | [4,6,7] |
| *Mergus merganser* | | 0.848 | | | 1.841 | [7] |
| *Mergus serrator* | | 0.713 | | | 1.823 | [4,6,7] |
| *Somateria mollissima* | | 0.905 | | | 2.033 | [11]; this study |
| *Tadorna cana* | | 0.898 | | | 1.919 | [4-6] |
| *Tadorna tadorna* | | 0.888 | | | 1.903 | [7]; this study |
| *Tadorna variegata* | | 1.095 | | | 1.952 | [4,6,7] |
| Charadriiformes | | | | | | |
| *Alca torda* | | | | 0.277 | 1.968 | [4-6] |
| *Brachyramphus marmoratus* | | | | 0.433 | 1.585 | [12,13] |
| *Burhinus oedicnemus* | | | | 0.677 | 1.525 | [5]; this study |
| *Cepphus columba* | | | | 0.589 | 1.756 | [12] |
| *Cerorhinca monocerata* | | | | 0.317 | 1.899 | [12,14] |
| *Fratercula arctica* | | | | 0.683 | 1.778 | [4-6]; this study |
| *Fratercula cirrhata* | | | | 0.703 | 1.954 | [6,12] |
| *Haematopus ostralegus* | | | | 0.256 | 1.618 | [4-6] |
| *Larus argentatus* | | | | 0.695 | 1.940 | [4-6] |
| *Larus canus* | | | | 0.593 | 1.699 | [4-6] |
| *Larus fuscus* | | | | 0.976 | 1.924 | [4-6] |
| *Larus glaucescens* | | | | 0.894 | 1.991 | [5]; this study |
| *Larus heermanni* | | | | 0.610 | 1.730 | [5,15]; this study |
| *Larus marinus* | | | | 0.878 | 2.057 | [4-6] |
| *Larus ridibundus* | | | | 0.300 | 1.555 | [5]; this study |
| *Numenius phaeopus* | | | | 0.774 | 1.728 | [4-6] |
| *Onychoprion fuscatus* | | | | 0.408 | 1.531 | [5]; this study |
| *Pluvialis apricaria* | | | | 0.294 | 1.513 | [4-6] |
| *Ptychoramphus aleuticus* | | | | -0.138 | 1.465 | [12] |
| *Rissa tridactyla* | | | | 0.393 | 1.699 | [5]; this study |
| *Rynchops niger* | | | | 0.043 | 1.422 | [5,16]; this study |
| *Stercorarius skua* | | | | 0.767 | 1.980 | [4-6] |
| *Sterna paradisaea* | | | | -0.319 | 1.255 | [5]; this study |
| *Sternula albifrons* | | | | -0.396 | 0.968 | [5]; this study |
| *Synthliboramphus antiquus* | | | | 0.060 | 1.651 | [12,17] |
| *Thalasseus elegans* | | | | 0.297 | 1.613 | [5]; this study |
| *Thalasseus maximus* | | | | 0.769 | 1.789 | [4,8]; this study |
| *Uria aalge* | | | | 0.749 | 2.048 | [6,12] |
| Ciconiiformes | | | | | | |
| *Egretta thula* | 0.333 | | | | 1.354 | [5]; this study |
| *Egretta tricolor* | 0.092 | | | | 1.407 | [5]; this study |
| *Eudocimus albus* | 0.213 | | | | 1.696 | [5]; this study |
| *Nycticorax nycticorax* | 0.147 | | | | 1.618 | [5]; this study |
| *Plegadis falcinellus* | 0.424 | | | | 1.571 | [5]; this study |
| Columbiformes | | | | | | |
| *Columba livia* | 0.050 | | | | 1.193 | [6,18] |
| *Streptopelia turtur* | -0.479 | | | | 0.919 | [5]; this study |
| Falconidae | | | | | | |
| *Falco naumanni* | -0.206 | | | | 1.035 | [5]; this study |
| *Falco tinnunculus* | -0.321 | | | | 1.258 | [4,6,19]; this study |
| Galliformes | | | | | | |
| *Alectura lathami* | | | 1.515 | | 2.299 | [2,5]; this study |
| *Ammoperdix heyi* | | | -0.652 | | 1.154 | [5]; this study |
| *Chrysolophus amherstiae* | | | 0.724 | | 1.484 | [5]; this study |
| *Coturnix coturnix* | | | 0.160 | | 0.982 | [4-6] |
| *Gallus gallus* | | | 0.273 | | 1.752 | [4,6,8] |
| *Leipoa ocellata* | | | 1.673 | | 2.197 | [1,4,6,20]; this study |
| *Lophophorus impejanus* | | | 0.337 | | 1.816 | [4-6] |
| *Lophura nycthemera* | | | 0.877 | | 1.601 | [4-6]; this study |
| *Megapodius decollatus* | | | 1.428 | | 2.069 | [21]; this study |
| *Meleagris gallopavo* | | | 0.741 | | 1.863 | [4,6,8] |
| *Numida meleagris* | | | 0.235 | | 1.689 | [6,22] |
| *Pavo cristatus* | | | 1.163 | | 1.978 | [5]; this study |
| *Phasianus colchicus* | | | 0.357 | | 1.498 | [4-6] |
| *Syrmaticus soemmerringii* | | | 0.436 | | 1.498 | [5]; this study |
| Passeriformes | | | | | | |
| *Agelaius phoeniceus* | -1.358 | | | | 0.633 | [23,24] |
| *Menura novaehollandiae* | 0.044 | | | | 1.789 | [3,25] |
| *Molothrus ater* | -1.154 | | | | 0.522 | [24,26] |
| *Passer domesticus* | -0.867 | | | | 0.441 | [5,6]; this study |
| *Spiza americana* | -1.682 | | | | 0.450 | [24,27] |
| *Turdus merula* | -0.278 | | | | 0.828 | [5,6,28] |
| Pelecaniformes | | | | | | |
| *Anhinga anhinga* | 0.469 | | | | 1.560 | [29]; this study |
| *Morus bassanus* | 0.397 | | | | 2.025 | [4-6] |
| *Phalacrocorax auritus* | 0.450 | | | | 1.696 | [5,6] |
| *Phalacrocorax carbo* | 0.392 | | | | 1.763 | [4-6] |
| *Phalacrocorax pelagicus* | 0.444 | | | | 1.598 | [5]; this study |
| Procellariiformes | | | | | | |
| *Diomedea exulans* | 1.357 | | | | 2.699 | [4-6] |
| *Fulmarus glacialis* | 1.129 | | | | 2.004 | [30]; this study |
| *Oceanodroma leucorhoa* | -0.295 | | | | 1.021 | [5]; this study |
| *Puffinus pacificus* | 0.844 | | | | 1.778 | [4-6] |
| *Puffinus puffinus* | 0.591 | | | | 1.766 | [6,31] |
| *Puffinus tenuirostris* | 0.927 | | | | 1.942 | [4,32] |
| Sphenisciformes | | | | | | |
| *Aptenodytes forsteri* | 1.188 | | | | 2.653 | [4-6] |
| *Aptenodytes patagonicus* | 1.149 | | | | 2.481 | [6,33] |
| *Eudyptes robustus* | 0.827 | | | | 2.065 | [34] |
| *Pygoscelis adeliae* | 0.899 | | | | 2.096 | [35]; this study |
| *Spheniscus demersus* | 0.692 | | | | 2.003 | [4-6]; this study |
| Strigiformes | | | | | | |
| *Strix aluco* | 0.207 | | | | 1.558 | [5]; this study |
| *Tyto alba* | 0.126 | | | | 1.272 | [5]; this study |
| Struthioniformes | | | | | | |
| *Apteryx australis* | 1.024 | | | | 2.544 | [36,37] |
| Tinamiformes | | | | | | |
| *Eudromia elegans* | 0.408 | | | | 1.554 | [5]; this study |
| Crocodylia | | | | | | |
| *Alligator mississippiensis* | 1.497 | | | | 1.859 | [38-40]; this study |
| *Alligator sinensis* | 1.121 | | | | 1.713 | [41,42]; this study |
| *Caiman crocodilus* | 1.175 | | | | 1.771 | [41]; this study |
| *Caiman latirostris* | 1.736 | | | | 1.829 | [43]; this study |
| *Caiman yacare* | 1.264 | | | | 1.877 | [41]; this study |
| *Crocodylus mindorensis* | 1.462 | | | | 1.867 | [40,44] |
| *Crocodylus moreletii* | 1.130 | | | | 1.839 | [45]; this study |
| *Crocodylus niloticus* | 1.300 | | | | 2.041 | [41]; this study |
| *Crocodylus porosus* | 1.325 | | | | 2.053 | [41]; this study |
| *Crocodylus rhombifer* | 1.325 | | | | 2.018 | [41,44]; this study |
| *Crocodylus siamensis* | 1.245 | | | | 2.029 | [41,44]; this study |
| *Gavialis gangeticus* | 2.080 | | | | 2.208 | [41,44]; this study |
| *Melanosushus niger* | 1.548 | | | | 2.157 | [41,44]; this study |
| *Osteolaemus tetraspis* | 0.766 | | | | 1.715 | [41]; this study |
| *Paleosuchus palpebrosus* | 1.522 | | | | 1.837 | [40,41]; this study |
| *Paleosuchus trigonatus* | 1.861 | | | | 1.858 | [46]; this study |
| *Tomistoma schlegelii* | 1.658 | | | | 2.146 | [41,44]; this study |

**References**

1. Booth DT, Seymour RS (1987) Effect of eggshell thinning on water vapor conductance of Malleefowl eggs. Condor 89: 453-459.

2. Booth DT, Thompson MB (1991) A comparison of reptilian eggs with those of megapode birds. In: Deeming DC, Ferguson MWJ, editors. Egg Incubation: Its Effects on Embryonic Development in Birds and Reptiles. Cambridge, United Kingdom: Cambridge University Press. pp. 325-344.

3. Lill A (1987) Water vapor conductance and shell characteristics of Superb Lyrebird eggs. Australian Journal of Zoology 35: 553-558.

4. Schonwetter M (1960-1967) Handbuch der Oologie. Berlin, Germany: Akademie Verlag. 929 p.

5. Ar A, Rahn H (1985) Pores in avian eggshells: gas conductance, gas exchange and embryonic growth rate. Respiration Physiology 61: 1-20.

6. Tullett SG (1976) The avian eggshell: a mediating boundary [Ph.D.]. Bath, United Kingdom: The University of Bath. 198 p.

7. Hoyt DF, Board RG, Rahn H, Paganelli CV (1979) The eggs of the Anatidae: conductance, pore structure, and metabolism. Physiological Zoology 52: 438-450.

8. Rokitka MA, Rahn H (1987) Regional differences in shell conductance and pore density of avian eggs. Respiration Physiology 68: 371-376.

9. Balkan M, Karakas R, Biricik M (2006) Changes in eggshell thickness, shell conductance and pore density during incubation in the Peking Duck (*Anas platyrhynchos* f. *dom*.). Ornis Fennica 83: 117-123.

10. Carey C, Leonvelarde F, Monge C (1990) Eggshell conductance and other physical characteristics of avian eggs laid in the Peruvian Andes. Condor 92: 790-793.

11. Rahn H, Krog J, Mehlum F (1983) Microclimate of the nest and egg water loss of the Eider *Somateria mollissima* and other waterfowl in Spitsbergen. Polar Research 1: 171-184.

12. Zimmermann K, Hipfner JM (2007) Egg size, eggshell porosity, and incubation period in the marine bird family. Auk 124: 307-315.

13. Nelson SK (1997) Marbled murrelet (*Brachyramphus marmoratus*). In: Poole A, Gill F, editors. The birds of North America: The Academy of Natural Sciences, Philadelphia, and The American Ornithologists' Unnion, Washington, D.C.

14. Hipfner JM, Charleton K, Davies WE (2004) Rates and consequences of relaying in Cassin's Auklets *Ptychoramphus aleuticus* and Rhinoceros Auklets *Cerorhinea monocerata* breeding in a seasonal environment. Journal of Avian Biology 35: 224-236.

15. Rahn H, Dawson WR (1979) Incubation water loss in eggs of Heermann's and Western Gulls. Physiological Zoology 52: 451-460.

16. Grant GS, Paganelli CV, Rahn H (1984) Microclimate of Gull-billed Tern and Black Skimmer nests. Condor 86: 337-338.

17. Gaston AJ (1994) Ancient murrelet (*Synthliboramphus antiquus*). In: Poole A, Gill F, editors. The birds of North America: The Academy of Natural Sciences, Philadelphia, and The American Ornithologists' Unnion, Washington, D.C.

18. Arad Z, Gavrieli-Levin I, Marder J (1988) Adaptation of the pigeon egg to incubation in dry hot environments. Physiological Zoology 61: 293-300.

19. Ar A, Rahn H (1978) Interdependence of gas conductance, incubation length, and weight of the avian egg. In: Piiper J, editor. Respiratory Function in Birds, Adult and Embryonic. Berlin, Germany: Springer-Verlag. pp. 227-235.

20. Booth DT (1987) Effect of temperature on development of Mallee Fowl *Leipoa ocellata* eggs. Physiological Zoology 60: 437-445.

21. Jones DN, Dekker RWRJ, Roselaar CS (1995) The Megapodes. Oxford, United Kingdom: Oxford University Press. 262 p.

22. Ancel A, Girard H (1992) Eggshell of the domestic Guinea Fowl. British Poultry Science 33: 993-1001.

23. Rahn H, Carey C, Balmas K, Bhatia B, Paganelli C (1977) Reduction of pore area of avian eggshell as an adaptation to altitude. Proceedings of the National Academy of Sciences of the United States of America 74: 3095-3098.

24. Jaeckle WB, Kiefer M, Childs B, Harper RG, Rivers JW, Peer BD (2012) Comparison of eggshell porosity and estimated gas flux between the brown-headed cowbird and two common hosts. Journal of Avian Biology 43: 486-490.

25. Higgins PJ, Peter JM, Steele WK (2001) Handbook of Australian, New Zealand and Antarctic Birds. Volume 5: Tyrant-flycatchers to Chats. Melbourne, Australia: Oxford University Press.

26. Ar A, Paganell CV, Reeves RB, Greene DG, Rahn H (1974) Avian egg: water vapor conductance, shell thickness, and functional pore area. Condor 76: 153-158.

27. Temple SA (2002) Dickcissel (*Spiza americana*). In: Poole A, Gill F, editors. The birds of North America, No 703. Philadelphia: The Birds of North America. pp. 1-24.

28. Higgins PJ, Peter JM, Cowling SJ (2006) Handbook of Australian, New Zealand and Antarctic Birds. Volume 7: Boatbill to Starlings. Melbourne, Australia: Oxford University Press. 1992 p.

29. Colacino JM, Hamel PB, Rahn H, Board RG, Sparks NHC (1985) The vaterite cover on the eggs of *Anhinga anhinga* and its effect on gas conductance (Aves: Anhingidae). Journal of Zoology 205: 425-433.

30. Rahn H, Greene DG, Toien O, Krog J, Mehlum F (1984) Estimated laying dates and eggshell conductance of the Fulmar and Brunnich's Murre in Spitsbergen. Ornis Scandinavica 15: 110-114.

31. Lee DS, Haney JC (1996) Manx Shearwater (*Puffinus puffinus*). In: Poole A, Gill F, editors. The Birds of North America, No 257: The Academy of Natural Sciences, Philadelphia, and the American Ornithologists' Union, Washington, D.C. pp. 1-28.

32. Fitzherbert K (1985) The role of energetic factors in the evolution of the breeding biology of the Short-tailled Shearwater (*Puffinus tenuirostris*, Temminck) [Ph.D.]. Victoria, Australia: Monash University. 225 p.

33. Handrich Y (1989) Incubation water loss in King Penguin egg .I. Change in egg and brood pouch parameters. Physiological Zoology 62: 96-118.

34. Massaro M, Davis LS (2005) Differences in egg size, shell thickness, pore density, pore diameter and water vapour conductance between first and second eggs of snares penguins *Eudyptes robustus* and their influence on hatching asynchrony. Ibis 147: 251-258.

35. Rahn H, Hammel HT (1982) Incubation water loss, shell conductance, and pore dimensions in Adelie Penguin eggs. Polar Biology 1: 91-97.

36. Calder III WA, Parr CR, Karl DP (1978) Energy content of eggs of Brown Kiwi *Apteryx australis*; extreme in avian evolution. Comparative Biochemistry and Physiology A 60: 177-179.

37. Silyn-Roberts H (1983) The pore geometry and structure of the eggshell of the North Island Brown Kiwi, *Apteryx australis mantelli*. Journal of Microscopy 130: 23-36.

38. Packard GC, Taigen TL, Packard MJ, Shuman RD (1979) Water vapor conductance of testudinian and crocodilian eggs (class Reptilia). Respiration Physiology 38: 1-10.

39. Wink CS, Elsey RM, Bouvier M (1990) Porosity of eggshells from wild and captive, Pen-reared Alligators (*Alligator mississippiensis*). Journal of Morphology 203: 35-39.

40. Marzola M, Russo J, Mateus O (2015) Identification and comparison of modern and fossil crocodilian eggs and eggshell structures. Historical Biology 27: 115-133.

41. Ferguson MWJ (1985) Reproductive biology and embryology of the crocodilians. In: Gans C, editor. Biology of the Reptilia. New York: John Wiley and Sons. pp. 329-491.

42. Wink CS, Elsey RM (1994) Morphology of shells from viable and nonviable eggs of the Chinese Alligator (*Alligator sinensis*). Journal of Morphology 222: 103-110.

43. Stoker C, Zayas MA, Ferreira MA, Durando M, Galoppo GH, Rodriguez HA, et al. (2013) The eggshell features and clutch viability of the Broad-snouted Caiman (*Caiman latirostris*) are associated with the egg burden of organochlorine compounds. Ecotoxicology and Environmental Safety 98: 191-195.

44. Thorbjarnarson JB (1996) Reproductive characteristics of the order Crocodylia. Herpetologica 52: 8-24.

45. Platt SG, Rainwater TR, Thorbjarnarson JB, McMurry ST (2008) Reproductive dynamics of a tropical freshwater crocodilian: Morelet's crocodile in northern Belize. Journal of Zoology 275: 177-189.

46. Rivas JA, Aktay SA, Owens RY (2001) *Paleosuchus trigonatus* (Schneider's Smooth-fronted Caiman) nesting. Herpetological Review 32: 251.
