# Supplementary material for: Eggshell Porosity Provides Insight on Evolution of Nesting in Dinosaurs
Source: PLoS One. 2015 Nov 25;10(11):e0142829. doi: 10.1371/journal.pone.0142829 (PMC4659668; doi:10.1371/journal.pone.0142829)
Supplement: S4 Table — (DOCX) [file pone.0142829.s009.docx]

**S4 Table. Nest type classification for living archosaur species.**

| Taxon | | Nest type | References |
| --- | --- | --- | --- |
| Accipitridae | *Buteo rufinus* | Open | [1,2] |
| Anseriformes | *Aix galericulata* | Open | [3,4] |
|  | *Aix sponsa* | Open | [4-7] |
|  | *Anas bahamensis* | Open | [4] |
|  | *Anas discors* | Open | [5] |
|  | *Anas fulvigula* | Open | [8,9] |
|  | *Anas gracilis* | Open | [10,11] |
|  | *Anas platyrhynchos* | Open | [4] |
|  | *Anser anser* | Open | [3,12] |
|  | *Anser brachyrhynchus* | Open | [4] |
|  | *Anser cygnoides* | Open | [4] |
|  | *Anser erythropus* | Open | [3] |
|  | *Anser fabalis* | Open | [3] |
|  | *Branta canadensis* | Open | [3] |
|  | *Branta h. minima* | Open | [3] |
|  | *Branta leucopsis* | Open | [3] |
|  | *Branta sandvicensis* | Open | [13,14] |
|  | *Bucephala islandica* | Open | [3] |
|  | *Cairina moschata* | Open | [5] |
|  | *Cereopsis novaehollandiae* | Open | [4,11] |
|  | *Chloephaga melanoptera* | Open | [4,15] |
|  | *Clangula hyemalis* | Open | [3] |
|  | *Cyanochen cyanoptera* | Open | [4,16] |
|  | *Dendrocygna arborea* | Open | [4] |
|  | *Dendrocygna autumnalis* | Open | [5] |
|  | *Dendrocygna bicolor* | Open | [5] |
|  | *Lophodytes cucullatus* | Open | [8,16] |
|  | *Mergus merganser* | Open | [3] |
|  | *Mergus serrator* | Open | [3] |
|  | *Somateria mollissima* | Open | [3,17] |
|  | *Tadorna cana* | Open | [4] |
|  | *Tadorna tadorna* | Open | [3] |
|  | *Tadorna variegata* | Open | [16,18] |
| Charadriiformes | *Alca torda* | Open | [5] |
|  | *Brachyramphus marmoratus* | Open | [5] |
|  | *Burhinus oedicnemus* | Open | [3] |
|  | *Cepphus columba* | Open | [5] |
|  | *Cerorhinca monocerata* | Open | [5] |
|  | *Fratercula arctica* | Open | [3] |
|  | *Fratercula cirrhata* | Open | [5] |
|  | *Haematopus ostralegus* | Open | [3] |
|  | *Larus argentatus* | Open | [3] |
|  | *Larus canus* | Open | [3,19] |
|  | *Larus fuscus* | Open | [3] |
|  | *Larus glaucescens* | Open | [5] |
|  | *Larus heermanni* | Open | [5] |
|  | *Larus marinus* | Open | [3] |
|  | *Larus ridibundus* | Open | [3,20] |
|  | *Numenius phaeopus* | Open | [3] |
|  | *Onychoprion fuscatus* | Open | [5] |
|  | *Pluvialis apricaria* | Open | [3] |
|  | *Ptychoramphus aleuticus* | Open | [5] |
|  | *Rissa tridactyla* | Open | [3,5] |
|  | *Rynchops niger* | Open | [5] |
|  | *Stercorarius skua* | Open | [3] |
|  | *Sterna paradisaea* | Open | [3,21] |
|  | *Sternula albifrons* | Open | [3] |
|  | *Synthliboramphus antiquus* | Open | [5] |
|  | *Thalasseus elegans* | Open | [5] |
|  | *Thalasseus maximus* | Open | [5] |
|  | *Uria aalge* | Open | [3] |
| Ciconiiformes | *Egretta thula* | Open | [5] |
|  | *Egretta tricolor* | Open | [5] |
|  | *Eudocimus albus* | Open | [5] |
|  | *Nycticorax nycticorax* | Open | [3] |
|  | *Plegadis falcinellus* | Open | [3] |
| Columbiformes | *Columba livia* | Open | [3] |
|  | *Streptopelia turtur* | Open | [3] |
| Falconidae | *Falco naumanni* | Open | [1,3,22] |
|  | *Falco tinnunculus* | Open | [3,22] |
| Galliformes | *Alectura lathami* | Covered | [11] |
|  | *Ammoperdix heyi* | Open | [3,22] |
|  | *Chrysolophus amherstiae* | Open | [1,23] |
|  | *Coturnix coturnix* | Open | [3] |
|  | *Gallus gallus* | Open | [11] |
|  | *Leipoa ocellata* | Covered | [11] |
|  | *Lophophorus impejanus* | Open | [23] |
|  | *Lophura nycthemera* | Open | [3] |
|  | *Megapodius decollatus* | Covered | [24] |
|  | *Meleagris gallopavo* | Open | [3] |
|  | *Numida meleagris* | Open | [5] |
|  | *Pavo cristatus* | Open | [1,25] |
|  | *Phasianus colchicus* | Open | [3] |
|  | *Syrmaticus soemmerringii* | Open | [26] |
| Passeriformes | *Agelaius phoeniceus* | Open | [5] |
|  | *Menura novaehollandiae* | Open | [11,27] |
|  | *Molothrus ater* | Open | [5] |
|  | *Passer domesticus* | Open | [3] |
|  | *Spiza americana* | Open | [28] |
|  | *Turdus merula* | Open | [3] |
| Pelecaniformes | *Anhinga anhinga* | Open | [3] |
|  | *Morus bassanus* | Open | [5] |
|  | *Phalacrocorax auritus* | Open | [5] |
|  | *Phalacrocorax carbo* | Open | [5] |
|  | *Phalacrocorax pelagicus* | Open | [5] |
| Procellariiformes | *Diomedea exulans* | Open | [16] |
|  | *Fulmarus glacialis* | Open | [3,12] |
|  | *Oceanodroma leucorhoa* | Open | [3] |
|  | *Puffinus pacificus* | Open | [29,30] |
|  | *Puffinus puffinus* | Open | [5] |
|  | *Puffinus tenuirostris* | Open | [31] |
| Sphenisciformes | *Aptenodytes forsteri* | Open | [16] |
|  | *Aptenodytes patagonicus* | Open | [32] |
|  | *Eudyptes robustus* | Open | [33] |
|  | *Pygoscelis adeliae* | Open | [34] |
|  | *Spheniscus demersus* | Open | [35,36] |
| Strigiformes | *Strix aluco* | Open | [3] |
|  | *Tyto alba* | Open | [3] |
| Struthioniformes | *Apteryx australis* | Open | [37,38] |
| Tinamiformes | *Eudromia elegans* | Open | [39,40] |
| Crocodylia | *Alligator mississippiensis* | Covered | [41] |
|  | *Alligator sinensis* | Covered | [41] |
|  | *Caiman crocodilus* | Covered | [41] |
|  | *Caiman latirostris* | Covered | [41] |
|  | *Caiman yacare* | Covered | [41] |
|  | *Crocodylus mindorensis* | Covered | [41] |
|  | *Crocodylus moreletii* | Covered | [41] |
|  | *Crocodylus niloticus* | Covered | [41] |
|  | *Crocodylus porosus* | Covered | [41] |
|  | *Crocodylus rhombifer* | Covered | [41] |
|  | *Crocodylus siamensis* | Covered | [41] |
|  | *Gavialis gangeticus* | Covered | [41] |
|  | *Melanosushus niger* | Covered | [41] |
|  | *Osteolaemus tetraspis* | Covered | [41] |
|  | *Paleosuchus palpebrosus* | Covered | [41] |
|  | *Paleosuchus trigonatus* | Covered | [41] |
|  | *Tomistoma schlegelii* | Covered | [41] |

**References**

1. del Hoyo J, Elliott A, Sargatal J (1994) Handbook of the Birds of the World. Volume 2: New World Vultures to Guineafowl. Barcelona, Spain: Lynx Edicions. 638 p.

2. Hayman P, Hume R (2007) Bird: the ultimate illustrated guide to the birds of Britain and Europe. London, United Kingdom: Octopus Publishing. 552 p.

3. Harrison C (1975) A field guide to the nests, eggs and nestlings of European birds: with North Africa and the Middle East. London, United Kingdom: Collins. 432 p.

4. Kear J (2005) Ducks, Geese and Swans. Oxford, United Kingdom: Oxford University Press. 832 p.

5. Baicich PJ, Harrison CJO (1997) A guide to the nests, eggs, and nestlings of North American birds. London, United Kingdom: Academic Press. 347 p.

6. Delacour J (1959) The Waterfowl of the World. Volume 3. London, United Kingdom: McMlix. 270 p.

7. Hepp GR, Bellrose FC (1995) Wood Duck (*Aix sponsa*) In: Poole A, Gill F, editors. The birds of North America, 169: The Academy of Natural Sciences, Philadelphia, and The American Ornithologists' Union, Washington, DC.

8. Bellrose FC (1976) Ducks, Geese, and Swans of North America. Harrisburg: Stackpole Books. 543 p.

9. Stieglit WO, Wilson CT (1968) Breeding Biology of Florida Duck. Journal of Wildlife Management 32: 921-934.

10. Serventy DL, Whittell HM (1962) Birds of Western Australia. Perth, Australia: Paterson Brokensha. 427 p.

11. Pizzey G (1980) A Field Guide to the Birds of Australia. Sydney, Australia: Collins. 460 p.

12. Cramp S, Simmons KEL (1977) Handbook of the Birds of Europe, the Middle East, and North Africa: the Birds of the Western Palearctic. Volume 1: Ostrich to Ducks. Oxford, United Kingdom: Oxford University Press. 722 p.

13. Merne OJ (1974) Ducks, Geese and Swans. New York: St. Martin's Press. 160 p.

14. Banko PC, Black JM, Banko WE (1999) Hawaiian Goose (Nene) (*Branta sandvicensis*). In: Poole A, Gill F, editors. The Birds of North America, 434: The Academy of Natural Sciences, Philadelphia, and The American Ornithologists' Union, Washington, DC.

15. Carey C, Leonvelarde F, Monge C (1990) Eggshell conductance and other physical characteristics of avian eggs laid in the Peruvian Andes. Condor 92: 790-793.

16. del Hoyo J, Elliott A, Sargatal J (1992) Handbook of the Birds of the World. Volume 1: Ostrich to Ducks. Barcelona, Spain: Lynx Edicions. 696 p.

17. Mehlum F (1991) Egg predation in a breeding colony of the Common Eider *Somateria mollissima* in Kongsfjorden, Svalbard. Norsk Polarinstitutt Skrifter 195: 37-45.

18. Williams M (1979) The social structure, breeding and population dynamics of Paraside Shelduck in the Gisborne-East Coast District. Notornis 26: 213-272.

19. Burger J, Gochfeld M (1987) Nest-site selection by Mew Gulls (*Larus canus*): a comparison of marsh and dry-land colonies. Wilson Bulletin 99: 673-687.

20. Cramp S, Simmons KEL (1983) Handbook of the Birds of Europe, the Middle East, and North Africa: the Birds of the Western Palearctic. Volume 3: Warders to Gulls. Oxford, United Kingdom: Oxford University Press. 1000 p.

21. Cramp S (1985) Handbook of the Birds of Europe, the Middle East, and North Africa: the Birds of the Western Palearctic. Volume 4: Terns to Woodpeckers. Oxford, United Kingdom: Oxford University Press.

22. Snow DW, Perrins CM (1998) The Birds of the Western Palearctic. Volume 1: Non-Passerines. Oxford, United Kingdom: Oxford University Press. 1008 p.

23. Beebe W (1931) Pheasants: Their Lives and Homes. Volumes 1 and 2. New York: Doubleday, Doran and Co. 566 p.

24. Jones DN, Dekker RWRJ, Roselaar CS (1995) The Megapodes. Oxford, United Kingdom: Oxford University Press. 262 p.

25. Whistler HFZS (1949) Popular Handbook of Indian Birds. Edimburgh and London, United Kingdom: Oliver and Boyd. 414 p.

26. Yamashina Y (1961) Birds in Japan: a Field Guide. Tokyo, Japan: Tokyo News Service. 233 p.

27. Lill A (1979) Nest inattentiveness and its influence on development of the young in the Superb Lyrebird. Condor 81: 225-231.

28. del Hoyo J, Sargatal J, Christie DA (2011) Handbook of the Birds of the World. Volume 16: Tanagers to New World Blackbirds. Barcelona, Spain: Lynx Edicions. 800 p.

29. Howell TR, Bartholomew GA (1961) Temperature regulation in nesting Bonin Island Petrels, Wedge-tailed Shearwaters, and Christmas Island Shearwaters. Auk 78: 343-354.

30. Whittow GC (1997) Wedge-tailed Shearwater (*Puffinus pacificus*). In: Poole A, Gill F, editors. The birds of North America, 305: The Academy of Natural Sciences, Philadelphia, and The American Ornithologists' Unnion, Washington, DC.

31. Bradley JS, Wooller RD, Skira IJ (2000) Intermittent breeding in the Short-tailed Shearwater *Puffinus tenuirostris*. Journal of Animal Ecology 69: 639-650.

32. Handrich Y (1989) Incubation water loss in King Penguin egg. I. Change in egg and brood pouch parameters. Physiological Zoology 62: 96-118.

33. Warham J (1974) The breeding biology and behaviour of the snares crested penguin. Journal of the Royal Society of New Zealand 4: 63-108.

34. Goodfellow P (1977) Birds as Builders. London, United Kingdom: David and Charles. 168 p.

35. Seddon PJ, van Heezik Y (1991) Effects of hatching order, sbling asymmetries, and nest site on survival analysis of Jackass Penguin chicks. Auk 108: 548-555.

36. Kemper J, Underhill LG, Roux J-P, Bartlett PA, Chesselet YJ, James JAC, et al. (2007) Breeding patterns and factors influencing breeding success of African Penguins *Spheniscus demersus* in Namibia. In: Kirkman SP, editor. Final Report of the BCLME (Benguela Current Large Marine Ecosystem) Project on Top Predators as Biological Indicators of Ecosystem Change in the BCLME. Cape Town, South Africa: Avian Demography Unit. pp. 89-99.

37. Calder III WA (1979) Kiwi and egg design: evolution as a package deal. BioScience 29: 461-467.

38. Colbourne R (2002) Incubation behaviour and egg physiology of Kiwi (*Apteryx* spp.) in natural habitats. New Zealand Journal of Ecology 26: 129-138.

39. Mezquida ET (2001) Ecologia reproductiva de un ensamble de aves del desierto del monte central, Argentina [Ph.D.]. Madrid, Spain: Universidad Autonoma de Madrid. 149 p.

40. Davies SJJF (2002) Ratites and Tinamous: Tinamidae, Rheidae, Dromaiidae, Casuariidae, Apterygidae, Struthionidae. Oxford, United Kingdom: Oxford University Press. 310 p.

41. Brazaitis P, Watanabe ME (2011) Crocodilian behaviour: a window to dinosaur behaviour? Historical Biology 23: 73-90.
